# The Neural Basis of Following Advice

**DOI:** 10.1371/journal.pbio.1001089

**Published:** 2011-06-21

**Authors:** Guido Biele, Jörg Rieskamp, Lea K. Krugel, Hauke R. Heekeren

**Affiliations:** 1 Max Planck Institute for Human Development, Berlin, Germany; 2 Center for the Study of Human Cognition, Department of Psychology, University of Oslo, Oslo, Norway; 3 Department of Education and Psychology, Freie Universität Berlin, Berlin, Germany; 4 Department of Psychology, University of Basel, Basel, Switzerland; 5 Department of Neurology, Charité University Medicine Berlin, Berlin, Germany; University of Oxford, United Kingdom

## Abstract

Learning by following explicit advice is fundamental for human cultural evolution, yet the neurobiology of adaptive social learning is largely unknown. Here, we used simulations to analyze the adaptive value of social learning mechanisms, computational modeling of behavioral data to describe cognitive mechanisms involved in social learning, and model-based functional magnetic resonance imaging (fMRI) to identify the neurobiological basis of following advice. One-time advice received before learning had a sustained influence on people's learning processes. This was best explained by social learning mechanisms implementing a more positive evaluation of the outcomes from recommended options. Computer simulations showed that this “outcome-bonus” accumulates more rewards than an alternative mechanism implementing higher initial reward expectation for recommended options. fMRI results revealed a neural outcome-bonus signal in the septal area and the left caudate. This neural signal coded rewards in the absence of advice, and crucially, it signaled greater positive rewards for positive and negative feedback after recommended rather than after non-recommended choices. Hence, our results indicate that following advice is intrinsically rewarding. A positive correlation between the model's outcome-bonus parameter and amygdala activity after positive feedback directly relates the computational model to brain activity. These results advance the understanding of social learning by providing a neurobiological account for adaptive learning from advice.

## Author Summary

Learning by following advice is fundamental for human cultural evolution. Yet it is largely unknown how the brain implements advice-taking in order to maximize rewards. Here, we used functional magnetic resonance imaging (fMRI) and behavioral experiments to study how people use one-off advice. We find that advice had a sustained effect on choices and modulated learning in two ways. First, participants initially assumed that the recommended option was most beneficial. Second, and more importantly, gains and losses obtained after following advice received an “outcome-bonus,” in which they were evaluated more positively than after not following advice. In other words, following advice was in general intrinsically rewarding. Computer simulations showed that the outcome-bonus is adaptive, because it benefits from good advice and limits the effect of bad advice. The fMRI analysis revealed a neural outcome-bonus signal in the septal area and left caudate head, structures previously implicated in trust and reward based learning. Participants with greater outcome-bonuses showed a greater gain-signal increase after following advice in the amygdala, a structure implicated in processing emotions and social information. In sum, these results suggest that decision makers adaptively combine advice and individual learning with a social learning mechanism in which advice modulates the neural reward response.

## Introduction

The nature and level of social learning in human societies is unmatched in the animal world. Especially when decisions are difficult, people rely on advice or recommendations regarding a decision or course of action [1]. Accumulating knowledge through social learning (particularly advice taking) is uniquely human and fundamental to the evolution of human culture [2]–[4], and it is plausible that genetic adaptations to social learning evolved in humans [5]. Cumulative social learning strongly relies on advice taking, which transmits social information more reliably than imitation or observational learning. For the individual, heeding advice can be especially useful when mistakes are costly and social information is accurate [4],[6]. Accordingly, advice taking affects many domains of learning and decision making, such as cooperation [6],[7], financial decisions [8], or consumer behavior [9]. For instance, people do not discover a healthy diet by trial and error but combine recommendations from others with their own experiences to choose their meals.

The influence of advice and social learning in general does not require direct personal interaction but can be observed in situations where social information is transmitted by observation or by written or spoken advice [10]–[12]. Recent fMRI experiments provided the first insights into the neurobiological mechanisms underlying social learning. Social prediction error signals are used to learn about the probability of good advice from advisors with sometimes cooperative and sometimes uncooperative motives [13] and determine to what extent initial judgments are adjusted based on social information [14]. However, these results do not provide a mechanistic explanation for the often-observed sustained influence of advice or, more generally, the human propensity for social learning. In particular, it remains unclear if and how the brain implements an adaptive social learning mechanism to combine supportive advice with individual information gained through personal experience.

Reinforcement learning models [15] can provide hypotheses about the influence of advice on decision making, especially when decisions are based on past experiences. These models specify distinct sub-mechanisms of learning, such as the initial evaluation of choice options or the repeated evaluation of choice outcomes, which have different behavioral [16] and neuronal signatures [17] that may be separately influenced by advice. Behavioral studies have shown that the human propensity for following advice could be explained by its influence on the evaluation of outcomes rather than on initial reward expectations or choice processes [12],[18]. This influence is described best by an outcome-bonus model [12], which postulates a learning mechanism in which a reward bonus is added to both good and bad outcomes of recommended options. More specifically, rewards from recommended options lead to stronger positive reinforcements than rewards with the same objective value from alternative, non-recommended options. Correspondingly, punishments from recommended options inhibit the choice of that option less than punishments with the same objective disutility from non-recommended options. In fact, when the punishment from a recommended option is smaller than the size of the outcome-bonus, the punishment may still lead to a positive reinforcement. The behavioral evidence in favor of the outcome-bonus model suggests that the neurobiological implementation of advice-following relies on the brain's reward system. Neurophysiological experiments in monkeys and fMRI experiments in humans [15],[19] consistently report reward representation in targets of dopaminergic midbrain projection neurons. Positive outcomes (rewards) elicit an increase in blood-oxygen level-dependent (BOLD) responses in the ventromedial prefrontal cortex (VMPFC) [20],[21], the medial prefrontal cortex (MPFC) [22],[23], the amygdala [24], and the ventral striatum (VST) [25]–[27]. Of particular interest in the context of social learning is the septal area, because it signals reward [28] and triggers release of oxytocin [29], which in turn is known to enhance trusting behavior [30]. Hence, we predicted that positive outcomes from a recommended decision would lead to greater positive BOLD responses than positive outcomes from non-recommended decisions in these reward sensitive regions. Furthermore, whereas negative outcomes should lead to a negative BOLD response after choosing a non-recommended option, negative outcomes after choosing a recommended option should lead to an attenuated BOLD response decrease or even to a positive BOLD response.

Based on these predictions, we investigated if and how the outcome-bonus is implemented in the brain. In addition, we compared computational models and used simulations to test whether the outcome-bonus model provides the best explanation of behavior and if it is an adaptive social learning mechanism. We show that, compared to alternative social-learning mechanisms, the outcome-bonus is more adaptive and can better account for the observed behavior. Moreover, we identified a neural outcome-bonus signal in the septal area and the left caudate.

## Results

### Sustained Influence of Advice Is Explained by the Outcome-Bonus

**Figure 1:**
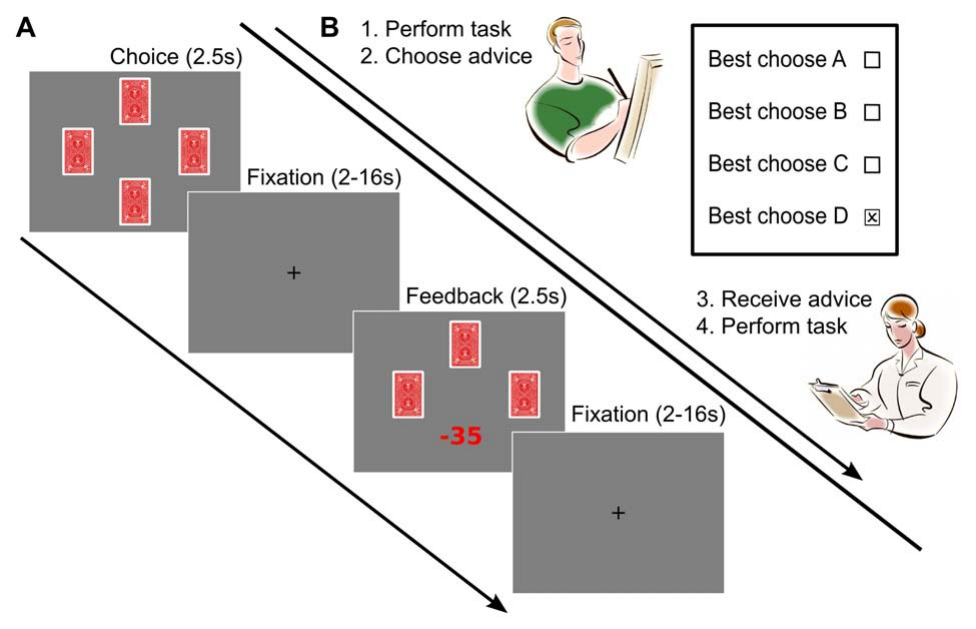
Experimental design. The task implemented key features of a realistic social learning situation, in which the learner receives well-intentioned advice prior to individual learning, but good advice is not certain. (A) In the learning task, participants repeatedly chose from one of four card decks associated with different gains and losses (c.f. Figure S1), with the goal of maximizing cumulative rewards. Each trial started with the presentation of the four decks. Participants had 2.5 s to choose a deck. After a variable fixation interval, feedback was presented for 2.5 s. (B) Advisors performed the 4-armed bandit task in the laboratory and indicated their advice on a form, which advice-receivers obtained before performing the task in the scanner.

**Figure 2:**
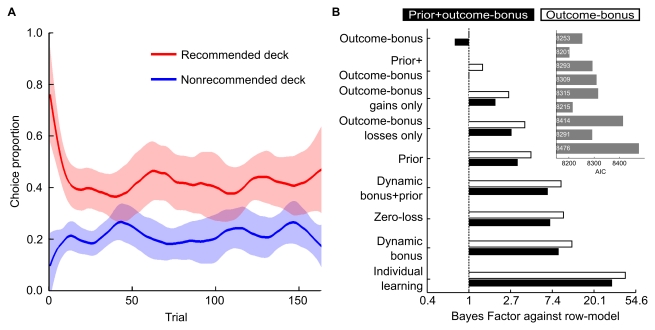
Behavioral and modeling results. (A) Participants' average choice proportions (running average of 11 trials ±2 standard errors) for the recommended and the non-recommended corresponding deck. This sustained influence of advice is consistent with the outcome-bonus mechanism, but not with the assumption that advice influences only the evaluation of choice options prior to individual learning (see below for detailed description of the social learning models). (B) The panel shows Bayes factors comparing the two models with the lowest Bayesian information criterion (outcome-bonus model and prior+outcome-bonus model) with the other models. The Bayes factor shows how much more likely the two best models are as compared to the model in the respective row. For instance, the last row shows that the prior+outcome-bonus model is about 30 times and the outcome-bonus model is about 40 times more likely than the individual learning model. Models assuming the influence of advice on evaluation of either gains or losses performed worse than the original outcome-bonus model. Moreover, more complex models implementing a dynamic outcome-bonus do not predict the data better than the simpler models with stable outcome-bonus. The inset (same order of models on the x-axis) shows that these results also hold when using the Akaike information criterion (AIC) as a model selection criterion. Because the AIC imposes smaller penalties for additional free parameters (for our sample size), models with a dynamic outcome-bonus perform better according to the AIC criterion. In sum, regardless of the model selection criterion, an outcome-bonus and a prior mechanism are implemented in the best models, but a dynamic or payoff-selective outcome-bonus mechanism is not.

Participants in the experiment learned that advice (i.e., a form on which the advisor marked which option the advice receiver should choose) was given from a second group of participants, who had previous experience with the task and were motivated to give good advice (see Figure 1 and Text S1 for details). Of the 21 participants, 16 received good advice. Regardless of good or bad advice, participants chose the recommended deck (41.5% of all choices) twice as often as they chose the non-recommended deck with the same payoff distribution (21.5% of all choices; *p*<.0001). Notably, this effect of advice was not limited to the beginning of the experiment, but rather was sustained; Figure 2A shows that participants robustly preferred the recommended deck to the non-recommended deck with the identical payoff distribution throughout the entire experiment. This result is consistent with the outcome-bonus but not with the assumption that advice influences only the evaluation of choice options prior to individual learning.

In the first half and, to a lesser extent, the second half of the trials, recipients of good advice chose the good decks more frequently than recipients of bad advice. The fact that this effect is greater in the first half (*p* = .039, effect size *r* = .39) than in the second half of the experiment (*p* = .137, effect size *r* = .25) indicates that bad advice harmed learning more during the first half of the experiment (c.f. Figure S2). The relatively weaker influence of bad advice in the second half of the experiment shows that decisions were made based on a combination of advice and individual learning, because only individual learning by the participants receiving bad advice can explain why they performed nearly as well as receivers of good advice in the second half of the experiment.

We evaluated the outcome-bonus model quantitatively by comparing it with alternative models based on standard model selection criteria. The results provide strong empirical support for the outcome-bonus mechanism as essential to explain social learning. In particular, we derived the Bayes factor from the Bayesian information criterion (BIC) [31]. With this model selection criterion, we found strong evidence in favor of the outcome-bonus model and a combined model implementing an outcome-bonus and higher initial reward expectations for the recommended deck (henceforth prior+outcome-bonus model). Figure 2B illustrates that, when considering the models' Bayes factors, these models predict the observed behavior equally well and much better than alternative models. Additionally, we compared the models by their Akaike information criterion (AIC) as an additional model selection criterion. Here, the prior+outcome-bonus model was the best model. Moreover, comparing the outcome-bonus, the prior, and the prior+outcome-bonus model against each other illustrates that removing the assumption of an outcome-bonus hurts the model fit more than removing the prior. When comparing the AIC (or BIC) values of the prior, the outcome-bonus, and the prior+outcome-bonus models with eight alternative models on a participant-by-participant level, the prior model is on average better for 59.7% (or 57.6%) of participants, the outcome-bonus model for 62.3% (or 64.4%), and the prior+outcome-bonus model for 67.1% (or 47.5%) (see Text S1 for details). Like the previous model comparison results, these comparisons underline the relevance of the outcome-bonus mechanism.

**Figure 3:**
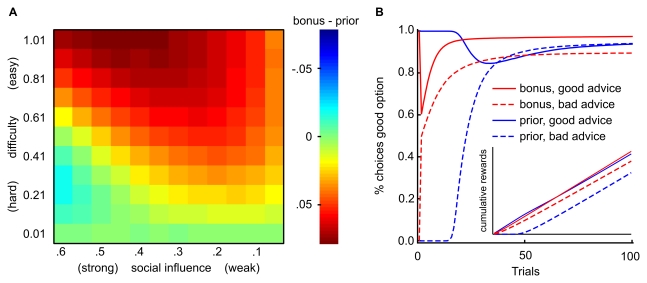
Adaptive value of social learning models. We used computer simulations to compare the average rewards gained by the prior model and the outcome-bonus model when performing a 4-armed bandit task with 100 trials after receiving advice about which bandit has the highest payoffs. To examine the models' performance over a range of learning settings, simulations were repeated for different task difficulties, levels of social influence, probabilities of correct advice, and learning parameters (see Text S1). (A) Difference in average payoffs across learning environments for 50% good advice. Each cell depicts the difference in average payoffs of the two models for a particular combination of task difficulty and social influence. The difference was computed from 1,000 simulated learning tasks for each model, whereby each model received good advice in half of the tasks. Difficulty, defined as difference in mean payoffs divided by payoff variance, is varied on the y-axis. Social influence, defined as either the magnitude of outcome-bonus (β_b_) or higher initial reward expectation for the recommended option (β_p_), is varied on the x-axis. The outcome-bonus model is more likely to choose a highly rewarded option (and thus accumulates most reward) for most levels of task difficulty and social influence, with exception of the combination of bad advice in a difficult task with strong social influence. (B) Learning curves show that the outcome-bonus model is better because it profits from good advice in the long run, even if individual learning already leads to a clear preference for a good bandit. The inset depicts cumulative rewards for both models. After bad advice, cumulative payoffs are reduced less for the outcome-bonus model compared to the prior model (see inset). In contrast, the prior model does not profit from good advice in the long run, and cumulative payoffs are greatly reduced after bad advice (see also Figures S3–S5). The inset also highlights that bad advice harms the prior model because it abolishes choices of the better option until the prior expectation induced by advice has been unlearned.

Simulated learning paths of the models illustrate that the outcome-bonus model, but not the prior model, predicts our key behavioral result, namely the sustained effect of advice on participants' behavior (c.f. Figures 2A and 3B). It might appear counterintuitive that the outcome-bonus is assumed to stay constant throughout the learning process. However, dynamic versions of the outcome-bonus model and the prior+outcome-bonus model, in which the outcome-bonus increases with time after good advice and decreases with time after bad advice, describe learning more poorly than the models using a constant outcome-bonus. Alternatively, advice could only influence the evaluation of gains or losses. These alternative models, however, again fitted the observed learning processes less well than the original outcome-bonus model (see Text S1 for details). In sum, regardless of the model selection criterion, the change of prior evaluations of options and more importantly the outcome-bonus mechanism are crucial constituents of descriptive social learning models for the influence of advice on learning. Because the prior+outcome-bonus model explained the data altogether best, we used predictions and parameters of this model in the fMRI analysis.

### The Outcome-Bonus Is an Adaptive Social Learning Mechanism

The outcome-bonus mechanism may be crucial to explain learning processes because it helps people to solve the learning task successfully. Indeed, when advice is more likely to be good than bad and the task is difficult and long (as was the case in our experiment), the outcome-bonus model is generally more adaptive (i.e., leading to higher average rewards) than the prior model because it ensures a lasting influence of good advice.

Beyond this basic insight, the simulation results depicted in Figure 3A show that, when good and bad advice are equally likely, the outcome-bonus model performs worse than the prior model only in a situation where at the same time (a) learning is difficult, and (b) the outcome-bonus is so large that recommended bad options are evaluated more positively than the objectively good options. Crucially, however, Figure 3A also shows that, in most situations, the outcome-bonus model outperforms the prior model when good and bad advice is equally likely. When advice is bad, the outcome-bonus model performs better because the prior model learns only late—after the wrong initial expectation for the recommended deck has been unlearned—which options are best (c.f. inset in Figure 3B). The advantage of the outcome-bonus model after bad advice is particularly strong for easier tasks where individual learning is relatively successful, because it does not interfere strongly with individual learning, whereas the prior does (see also Text S1). When advice is good, the outcome-bonus model performs better because it leads to a preference for a good option long after the effect of higher initial expectations has decayed (c.f. Figure 3B).

The superiority of the outcome-bonus model is notable, as the prior model resembles more a Bayesian approach, in which advice as prior information should influence the initial evaluation of choice options. However, whereas the prior model learns the expected values more accurately in the long run, cumulative rewards do depend on the choices made based on the learned values. The sustained overestimation of the rewards from a good option implemented by the outcome-bonus model (after good advice) helps to make the choice of that option occur more frequently and ultimately helps to accumulate more rewards.

### Following Advice Modulates BOLD Responses in the Reward System

**Figure 4:**
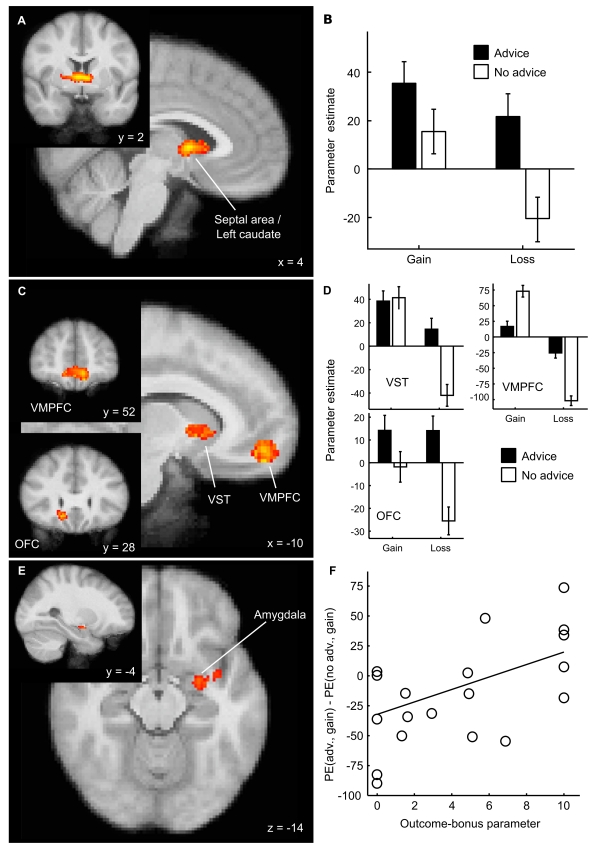
Neural basis of an outcome-bonus after following advice. (A) Feedback after following advice led to greater activity in the left caudate head and septal area. (B) Parameter estimates (PE) of the General Linear Model analysis suggest that the outcome-bonus is implemented in the septal area and left caudate as a greater increase in BOLD signal for gains and as a smaller decrease in BOLD signal for losses after following advice. (C and D) VST, VMPFC, and OFC showed a greater reduction in BOLD response to negative outcomes if these followed deviation from advice. (E) Participants with higher outcome-bonus parameters showed greater increases in BOLD signal in the left lateral amygdala for gains after following advice compared to after not following advice. (F) Scatter plot illustrating this correlation (1 outlier participant removed). Note that all regions implementing the outcome-bonus are gain preferring after not following advice, in that they all show an increase in BOLD signal to gains and a decrease in BOLD signal to losses.

We used fMRI to test the prediction that rewards from recommended versus non-recommended options would lead to greater BOLD responses in reward-sensitive brain regions. Regions implementing the outcome-bonus (outcome-bonus regions) should fulfill two conditions. First, when advice is followed, gains should lead to a greater increase in BOLD signals and losses should lead to a smaller decrease in BOLD signals (compared to when not following advice). Second, when advice is not followed, outcome-bonus regions should be gain-preferring; that is, these regions should show a regular reward signal with an increased positive BOLD signal after gains and a reduced BOLD signal after losses [32]. Only one cluster comprising the septal area and the left caudate head showed the predicted effect of advice (max. *z*-score  = 3.49; Montreal Neurological Institute [MNI] coordinates: *x* = 4, *y* = 2, *z* = 4; see Figure 4A and 4B) and was also gain-preferring, suggesting that this region implements the outcome-bonus. This outcome-bonus implementation cannot be explained in terms of different payoff distributions of the good and bad options because the experiment was designed such that good and bad decks were equally likely to lead to gains or losses (although the magnitudes of gains and losses differed). Moreover, because our fMRI analysis controlled for both different gain and loss magnitudes of good and bad options as well as different prediction error magnitudes of the advice and no-advice condition, the result can neither be ascribed to the fact that advisors recommended good options more frequently than bad options nor to differences in prediction errors elicited by feedbacks from the different choice options.

For more detailed insights into how the brain evaluates outcomes that are dependent on advice, we contrasted feedback-related BOLD responses separately for gains and losses in reward signaling regions after following and not following advice (see Figure 4C and 4D, and Text S1). For losses, we found greater BOLD responses after following advice in two gain-preferring regions: the VMPFC (max. *z*-score  = 3.35; *x* = −10, *y*  =  52, *z* = −18) and the left caudate (max. *z*-score  = 3.23; *x* = −16, *y* = 20, *z* = −6). However, these regions did not provide a complete outcome-bonus signal because the BOLD response to positive feedback was not greater after following advice. One cluster in the orbitofrontal cortex also showed a weaker BOLD signal reduction for losses after following advice (max. *z*-score  = 3.35; *x* = 16, *y* = 28, *z* = −12), but voxels in this region were not gain-preferring. For gains, we found that the difference between activity in the left amygdala after following or not following advice correlates with the outcome-bonus parameter of the prior+outcome-bonus model (max. *z*-score  = 3.02; *x* = −26, *y* = −4, *z* = −14), suggesting that the amygdala also implements the outcome-bonus.

**Figure 5:**
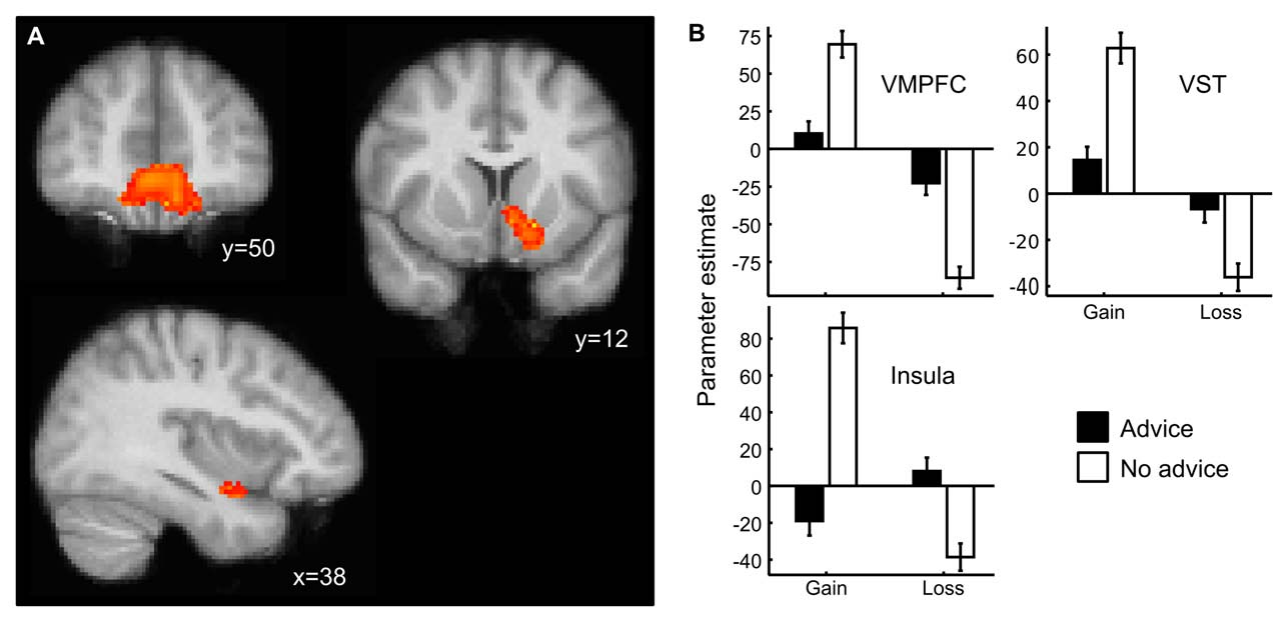
Advice modulated the standard BOLD response to rewards. (A) Regions showing greater BOLD response to positive than to negative feedback after not following advice. Top left: VMPFC (max. *z*-score = 3.50, *x* = −4, *y* = 50, *z* = −18); top right: Left VST (max *z*-score  = 3.73, *x* = −16, *y* = 12, *z* = −10); bottom left: Insular cortex (max *z*-score  = 3.33, *x* = 38, *y* = 8, *z* = −16). (B) Estimated parameter estimates show that following advice led to an attenuation of the standard reward signal (as identified from trials in which participants did not follow advice) in the VMPFC and the VST.

To investigate how advice modulates standard brain responses to rewards, we investigated advice-dependent changes in brain regions that showed greater activity after not following advice for gains compared to losses. Such reward signals were identified in the VMPFC, the ventral striatum (VST), and the right insula. The parameter estimates of these regions for gains and losses after following and not following advice show that advice led to an attenuation of the BOLD response in the VMPFC and VST, such that gain and loss signals are closer to the baseline BOLD response after advice was followed (see Figure 5).

To check the robustness of the neural outcome-bonus signal resulting from the effect of advice, we performed supplementary fMRI analyses. First, the above described analysis did not reveal a correlation between BOLD responses and prediction errors, likely because it included separate regressors for positive versus negative payoffs, which captured the variance associated with positive versus negative prediction errors. Indeed, a supplementary fMRI analysis tailored to reveal a prediction error signal identified correlations with prediction errors in the VST (Figure S6A). Importantly, this analysis also revealed the above reported effect of advice on reward signals in the septal area and the left caudate head (Figure S6B). Second, to further investigate the existence of a sustained effect of advice on learning and the neural correlates underlying this effect, we performed another fMRI analysis that tested whether the outcome-bonus changed from the first to the second half of the trials in which advice was followed. Consistent with our modeling results showing that models with a dynamic outcome-bonus do not explain behavior substantially better than models with a constant outcome-bonus, we did not find a change in the neural outcome-bonus signal in the septal area over time. However, we found reduced BOLD responses for feedback after following advice in the paracingulate gyrus and the superior temporal sulcus (see Figures S7 and S8 for details), which are commonly associated with theory-of-mind processes and trusting behavior [33],[34].

## Discussion

Taken together, behavioral, modeling, simulation, and neuroimaging data provide strong convergent evidence for a sustained effect of well-intentioned advice on decision making, which can be explained by an outcome-bonus model for following advice. Behavioral data showed that advice had a long-lasting influence on decision making and learning. Simulations suggest that the outcome-bonus is an adaptive social learning mechanism in a broad range of social learning environments. The model comparison showed that the outcome-bonus is necessary to explain the behavioral effect of advice. fMRI data supported this conclusion, as advice modulated reward-related brain activity so that the gain-sensitive septal area and the left caudate head showed a greater reward signal after following rather than not following advice; even negative outcomes elicited a positive reward response when advice was followed.

One feature of the experiment was that participants controlled when to follow advice, so that advice-following trials were not randomized across the experiment. Hence, additional factors might have influenced the observed differences between following and not following advice. Future experiments that randomly interleave trials of tasks with and without advice should further investigate this issue.

Still, the current experiment allowed us to rule out a number of alternative mechanisms that could a priori explain advice following. Among these, the brain could provide a greater expected reward signal for the recommended option. Alternatively, choosing the non-recommended option could be associated with anticipated regret, or negative feedback for the recommended option could lead to particularly strong regret. Moreover, outcomes from the recommended option could be processed with greater attention. Our behavioral and fMRI results do not support these alternative hypotheses. First, the decision phase was not characterized by a greater reward anticipation signal in the VST or the VMPFC when participants chose the recommended deck. Rather, the change in BOLD signal in a number of brain regions was smaller when choices were made and advice was followed (see Figure S9). This replicates the findings of an earlier study, which examined the effect of advice on investment decisions [35] and reported reduced activity in decision-related regions during advice trials. We did not find a greater change in the BOLD signal in regret-associated regions like the anterior cingulate cortex or the orbitofrontal cortex [36] during the choice or during the processing of negative feedback from non-recommended decks. Our fMRI results are also inconsistent with an attention account as we found that the reward signal in the VMPFC, as identified by contrasting gains and losses after not following advice, had a greater magnitude after not following compared to following advice. Similar results were reported for a study that compared orbitofrontal reward signals of self-determined and instructed choices [37]. Finally, simple attention effects cannot be reconciled readily neither with our behavioral finding that participants still learn which of the non-recommended decks is better nor with the notion that they prefer the recommended to the non-recommended of two options with the same expected value. Instead, our results suggest that advice modulates reward processing in two ways. First, the standard reward signal in the VMPFC and VST is attenuated. Second, the septal area and the left caudate head implement an outcome-bonus for recommended options. Importantly, the outcome-bonus signal does not replace the standard reward signal but seems to influence learning in addition to an attenuated standard reward signal.

Prior neuroimaging research on decision making in social contexts addressed the differences between social and nonsocial cognition [38] and the computational processes underlying decision making and learning in a social context [39]. Notably, recent studies showed that a social prediction error signal predicts future conformity with humans and computers [14] and that, when advice is given on a trial-by-trial level during strategic interaction, the brain tracks the quality of advice through social reinforcement learning signals [13].

We discovered that, on a neurobiological level, the human propensity for following trustworthy advice could be explained by the modulation of the neural reward response. Importantly, the outcome-bonus does not replace the standard reward signal. Instead, it supplements a still present, though attenuated, learning signal in the VMPFC and the VST (where a partial outcome-bonus is implemented). More specifically, only the septal area and the left caudate implement the full outcome-bonus signal. These regions signaled a more positive evaluation of outcomes after following advice and were also sensitive to rewards after not following advice. Notably, the septal area is ideally suited as the neural substrate of the outcome-bonus, because it contains neurons that mediate reinforcement [28] and project to nuclei in the hypothalamus that release oxytocin [29], a neurotransmitter known to facilitate trust [30]. Accordingly, a recent study showed greater activity in the septal area during trusting behavior [34]. Hence, our findings suggest that an intrinsic reward signal in the septal area facilitates trust, which, in turn, would facilitate future advice-following. The correlation of the outcome-bonus estimated for individual participants and the difference of positive reward signals in the amygdala after following versus not following advice suggests that this structure is also involved in maintaining the influence of advice. This result is plausible, as the amygdala is known to be involved in the detection of trust from faces [40] during social interaction [41] and in the generation of reward prediction errors during learning [39],[42].

The notion of intrinsic reward for following advice may seem counterintuitive, particularly because a Bayesian approach would suggest that advice influences expectations prior to individual experience. From an adaptive perspective, the relevant criterion to choose a social learning mechanism is the amount of reward that can be accumulated using a specific mechanism. Bayesian models are optimal in the sense that they accurately learn expected payoffs. This does not imply, however, that these models also accumulate most rewards because the obtained rewards depend also on how choices are derived from expected payoffs. Hence, when advice is predominantly good, the outcome-bonus model performs well as it biases choices persistently in the direction of the recommended option, whereas the prior model affects choices only initially.

Another interesting result is that the models implementing a dynamic outcome-bonus did not explain participants' behavior as well as the models implementing a stable outcome-bonus. We ascribe this to characteristics of our task designed to mimic everyday situations of advice following, in which the task at hand is often difficult, and the recommendation comes from a competent and motivated advisor. Hence, future research is needed to show whether the influence of advice is stable or dynamic when the task is relatively easy or the competence of the advisor is less uncertain.

The notion of intrinsic reward for following advice is consistent with both a learning and an evolutionary perspective. The effect of advice on reward representation suggests that following advice acts similarly to a secondary reinforcer. Following advice alone, which is usually followed by positive outcomes, elicits a reward response. Likewise, it has been proposed that imitation—another form of social learning—has the quality of a secondary reinforcer for children, who frequently experience that imitation leads to positive outcomes [43]. Mathematical analysis shows that the propensity for social learning can evolve on the population level in the environmental conditions that characterized the era of human evolutionary adaptation [2],[4]. Moreover, social learning can solve problems that individual learning cannot, such as cooperation in social dilemmas or the accumulation of knowledge across generations [2],[6],[7],[44]. Therefore, it seems plausible that humans have evolved mechanisms for social learning [5],[45]. We complement these explanations by providing a neurobiological account of an adaptive social learning mechanism, which can also explain the human propensity for social learning. Importantly, insights into the neurobiological mechanisms underlying social learning can pave the way for a targeted search of genetic adaptations to social learning. Based on our results, one could speculate that genetic adaptation to social learning involves genes that modulate reward processing.

In conclusion, we present evidence that the brain's reward system implements an adaptive social learning mechanism by generating a greater reward signal for outcomes received after following trustworthy advice. This outcome-bonus could also explain maladaptive social learning, which should occur particularly when the difference between choice options is hard to detect or when social influence is strong. Indeed, others have reported that decision makers will follow advice that implies sub-optimal decisions when decisions are difficult or contain a dilemma [7] and that social influence determines preferential choice beyond the quality of consumed goods [46]. Fundamentally, our results advance the understanding of social learning by providing a neurobiological account of the human propensity for social learning and of the sustained influence of social information on learning and decision making.

## Materials and Methods

### Participants

Twenty-one right-handed healthy participants performed a four-armed bandit task with 168 trials while being scanned in an MRI scanner. All participants were free of neurological and psychiatric history and gave written informed consent in accord with local ethics. An additional 10 participants were recruited to function as advisors for participants in the fMRI experiment.

### Task

Participants in the fMRI experiment received advice from a randomly selected advisor before entering the MRI scanner. To establish incentives for following advice, we truthfully informed participants that the advisor had performed the same task before and that the advisor's payment partially depended on the receiver's earnings. This design comes close to natural settings of advice-giving and -taking, where the advisor is motivated to give good advice, but the advice-receiver still cannot be entirely sure whether she receives the best advice.

In the learning task (performed in the MRI scanner), participants repeatedly chose from four card decks and received feedback after each trial (Figure 1 and Text S1). The four decks were comprised of two identical “good decks” with a high positive expected value and two identical “bad decks” with a low positive expected value (see Figure S1). Therefore, preference for the recommended deck over the corresponding deck with the same payoff distribution would be a clear indicator of the influence of advice. To examine the effect of advice on rewards and punishments, each card deck generated 50% positive and 50% negative payoffs across all trials. The bad decks had slightly higher gains but much larger losses than the good decks.

### Social Learning Models

To investigate the influence of advice on learning, we first compared how a standard reinforcement learning model, an “outcome-bonus” model, a “prior” model, and a combined “prior+outcome-bonus” model described participants' choices.

The standard reinforcement learning model assigns each option *i* an expected reward *q_i_*(*t*). On the basis of the expected rewards, choices are made according to the softmax choice rule [47], which determines the probability p*_i_*(*t*) of choosing option *i* of the *J* options in round *t* as follows:
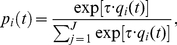
(1)where τ is a sensitivity parameter determining how likely the option with the largest expected reward will be chosen. Note that this choice function holds for all trials except the first, for which we assumed that the decision maker chooses the recommended option. This assumption was implemented in all tested models.

After a choice is made, the expected rewards are updated on the basis of the prediction error. That is, the deviation between the expected and actually received reward:
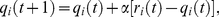
(2)where *r_i_*(*t*) is the reward obtained from choosing deck *i* in trial *t* and *α* is the learning rate that determines the impact of the prediction error in the updating process.

The outcome-bonus model differs from the standard reinforcement-learning model by changing the reinforcement of outcomes from recommended options. Accordingly, the updating rule (Equation 2) was modified such that when the recommended option was chosen, a constant bonus was added to the objective reward:
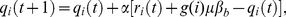
(3)where *g*(*i*) is an indicator function that takes the value 1 if option *i* is recommended and the value 0 if option *i* is not recommended, *β_b_* is a free outcome-bonus parameter capturing the level of social influence, and *μ* is the expected payoff from choosing randomly among all options and serves as a normalization constant to allow for comparison across tasks with different payoff magnitudes.

The prior model assumes a higher initial reward expectation for the recommended choice option. Hence, the initial reward expectation in the prior model is defined as 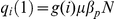, where *β_p_* captures the social influence on the prior expectations and *N* is the number of trials in the learning experiment, which we chose as a simple scaling factor, allowing for the comparison of the weight of the prior compared to the payoff that can be obtained in the experiment. For the combined prior+outcome-bonus model, both the initial reward expectation and the outcome-bonus were used to modify the evaluation of the choice options.

Additionally to the aforementioned models, we examined (a) a dynamic version of the outcome-bonus that becomes increasingly large after good advice and increasingly small and negative after bad advice. We also tested various other modifications of the outcome-bonus model, which (b) combined dynamic outcome-bonus and higher prior reward expectation for the recommended option, restricted the outcome-bonus to only (c) gains or (d) losses, (e) assumed that losses after following advice are processed as zero payoffs (see Text S1 for details).

For all models, we estimated the parameter values that maximized the log likelihood of trial-by-trial choice predictions for each participant separately (see Text S1). Model comparison was performed based on AIC and BIC values, which are derived from the log likelihood but additionally penalize models with a greater number of free parameters.

### fMRI Analysis

The functional analysis was based on 12 regressors (plus six motion-parameter regressors): Two regressors modeled the choice of the recommended and the non-recommended option(s), respectively. Four binary regressors modeled (a) positive and (b) negative feedback after choosing the recommended option and (c) positive and (d) negative feedback after non-recommended options, respectively. An additional set of four corresponding parametric regressors controlled for feedback magnitude. One regressor modeled prediction errors estimated with the combined prior+outcome-bonus model. One error regressor modeled feedback after missed trials, in which participants made no decisions. For group-level results, individual-level contrasts were averaged using the FMRIB Local Analysis of Mixed Effects module in FSL (see Text S1), and one-sample *t* tests were performed at each voxel for each contrast of interest.

To identify regions implementing advice and reward-sensitive feedback signals, we used the four regressors (a) through (d), described above. Advice-sensitive regions were identified by the contrast [1 1 −1 −1] for these regressors. To test if the resulting functional regions of interest (ROIs) were also reward-sensitive, we tested these voxels with the contrast [0 0 1 −1], based on the assumption that, after not following advice, feedback allows for the identification of reward responses that are uncontaminated by advice. An additional whole brain contrast, comparing gains and losses after not following advice [0 0 1 −1], revealed commonly reported reward signals in the VST and the VMPFC.

Following our predictions, we investigated representations of reward in a set of anatomically defined regions comprising the major reward-representing areas of the brain. We defined the reward ROIs based on the Harvard*-*Oxford subcortical structural atlas and included the following anatomical regions: nucleus accumbens, caudate, putamen, thalamus, medial frontal cortex, and amygdala. For the amygdala, *Z* (Gaussianized *T*) statistic images were thresholded with a small volume correction determined by *z*>2.576, and a minimum cluster size of 36 voxels determined with the AFNI AlphaSim tool (see Text S1). For the ROI comprising all other regions, *Z* statistic images were thresholded with a small volume correction determined by *z*>2.576 and a minimum cluster size of 92 voxels, also determined with the AFNI AlphaSim tool.

## Supporting Information

Figure S1 The payoff distribution for the four decks in the task. Good decks with higher average rewards were associated with relatively low gains and with moderate losses. Bad options with lower average rewards were associated with higher gains than the good decks, but also with much larger losses.
(TIF)

Figure S2 The probability of choosing one of the two good decks, separately for the first and second half of the experiment and for participants who received good and bad advice. Black lines indicate two standard errors of the mean. Participants who received bad advice (*n* = 5) generally chose the good decks less frequently than participants who received good advice (*n* = 16) but also improved performance from the first to the second half.
(TIF)

Figure S3 Performance of social learning models for typical learning parameters. Each panel shows one model's mean cumulative payoff on the *z*-axis. The levels of social influence and task difficulty are varied across the *x*- and *y*-axis, respectively. Difficulty is calculated as the difference in the mean payoff of the good and bad options, so that higher values indicate easier tasks. The performance of the outcome-bonus model is depicted by the blue surface, and the performance of the prior model is depicted by the red surface.
(TIF)

Figure S4 Performance of social learning models for typical learning parameters. (This figure presents the same data as Figure S3 in a different format.) Each subplot shows average payoffs of the two social learning models for different levels of social influence. The difficulty of the basic learning task (higher values indicate easier tasks) is varied along the global *y*-axis with easy tasks at the top and hard tasks at the bottom. The three columns show results for different qualities of advice. In sum, the figure indicates that the outcome-bonus model generally performs better. The prior model only performs better when, at the same time, (a) the task is hard, (b) bad advice is more likely, and (c) the social influence is relatively strong. The reason is that, in this case, the outcome-bonus can be larger than the payoff difference between good and bad options, so that the outcome-bonus model consistently prefers the recommended but bad option.
(TIF)

Figure S5 Performance differences in the social learning models across different learning parameters. Panel A shows the performance differences of the models. That is, the mean payoff of the outcome-bonus model minus the mean payoff of the prior model for typical learning parameters and 50% correct advice, while varying the impact of social influence (*x*-axis) and difficulty (*y*-axis). Panels C shows replications of the different difficulty and social learning parameters examined in Panel A for different learning rates (on the *x*-axis) and choice sensitivities (on the *y*-axis). Panels B and D show results of the same analysis for 25% and 75% good advice. Note that hotter colors (red and yellow) indicate an advantage of the outcome-bonus model, cooler colors (blue and cyan) indicate an advantage of the prior model, and neutral color (green) indicates similar performance of the two models.
(TIF)

Figure S6 The results of an alternative fMRI analysis optimized to detect correlations of BOLD response and prediction errors. The left panel shows a region in the ventral striatum that correlates positively with prediction errors for recommended and non-recommended options (x = −10, y = 6, z = −8, max *z*-value = 3.1). The right panel shows the results of contrasting the intercept regressors for recommended minus non-recommended feedback (x = −4, y = 10, z = 4, max *z*-value = 3.2). The effect of advice on feedback signals identified in this analysis comprises the same region as identified in the original analysis and reported in the main text.
(TIF)

Figure S7 The results of a supplementary analysis, investigating potential changes in the outcome-bonus signal over time. This analysis was based on the original analysis, to which we added two more regressors capturing (f) gain feedback- and (g) loss feedback-related activity after following advice for the second half of trials, in which participants chose the recommended option. Importantly, in this analysis, we found the same outcome-bonus signal in the septal area as identified in the original analysis (shown in Figure S8). Parameter estimates for regressors (f) and (g) did not differ significantly from zero in regions implementing the outcome-bonus. Instead, we found a reduced BOLD signal for feedback in regions commonly associated with theory-of-mind (TOM) reasoning or negative feedback after following advice. These results are significant after whole brain correction, based on a *z*-threshold of 2.576 and a minimum cluster size of 152 voxels (cluster size criterion obtained with AFNI AlphaSim, see Text S1). (A) Reduced activation for feedback after following advice (i.e. the contrast vector had a −1 for regressors (f) and (g) and 0 for all other regressors) in the dorsomedial prefrontal cortex/paracingulate cortex (DMPFC/PCC, x = −6, y = 42, z = 16, max *z*-value = 3.13). (B) Reduced activation for feedback after following advice in the superior temporal sulcus (STS, x = −64, y = −8, z = −6, max *z*-value  = 3.21). The PCC and STS are commonly associated with reasoning about the intentions and traits of other people and have been shown to be active when participants play economic games like the trust game [48],[49]. (C) Reduced activation for positive feedback after advice-following in the STS (x = −62, y = −6, z = −4, max *z*-value  = 3.11). (D) Reduced activation for negative feedback after following advice in the rostral cingulate zone (RCZ, x = −20, y = 24, z = 48, max *z*-value  = 3.21). The RCZ is associated with processes of conflict monitoring and learning from negative feedback [50]. (E) Reduced activation for negative feedback after following advice in the anterior cingulate cortex (ACC, x = −2, y = 2, z = 32, max *z*-value  = 3.21). In sum, these fMRI results suggest that the neural outcome-bonus does not change over the course of learning. Note that this is consistent with our finding that the dynamic outcome-bonus models do not fit participants' behavior better than the models with a constant outcome-bonus. Furthermore, these results are consistent with the hypothesis that participants reason more about the advisors' intentions and capabilities during the first half of the experiment and that they experience negative feedback as less conflicting after following advice in the later stages of the experiment (possibly because they have learned that losses cannot be avoided).
(TIF)

Figure S8 Result for the contrast of feedback advised > feedback not advised of the control analysis described in the legend to Figure S7. We still identified an outcome-bonus signal for feedback after following advice versus not following advice in the septal area/left caudate (x = −6, y = 12, z = 6, max *z*-value  = 3.26) over the entire experiment, after controlling for the effects of time by introducing additional regressors modeling feedback effects for the second half of the experiment (details in the legend to Figure S7).
(TIF)

Figure S9 A number of regions showed greater activation when participants decided to not follow advice. (A) Left supplementary motor area (SMA, x = −28, y = −6, z = 60, max *z*-value  = 3.33), (B) right post-central gyrus (x = 46, y = −34, z = 60, max *z*-value  = 3.56), (C) left central operculum with extension into caudate and putamen (x = −34, y = 8, z = 10, max *z*-value  = 3.05), (D) and left VST (x = −28, y = −6, z = 60, max *z*-value  = 3.07). Results shown in (A, B, C) are significant after whole brain correction, based on a *z*-threshold of 2.576 and a minimum cluster size of 152 voxels (cluster size criterion obtained with AFNI AlphaSim). With a cluster size of 81 voxels, the VST result approaches significance when correcting for multiple comparison in the reward ROI (minimum cluster size would be 92 voxel). In particular, the reduced activation in the SMA and the VST suggests that following advice is accompanied by a reduction in decision-related brain activity. A similar result has been reported for following advice in the context of financial decisions [35].
(TIF)

Table S1 The table provides basic information about the compared models. Columns under the header “Implemented social learning mechanisms” contain a “+” if a particular model implemented the respective social learning mechanism. LL is the log likelihood, AIC is the Aikake information criterion, BIC is the Bayesian information criterion (see supplementary methods for details).
(DOC)

Table S2 Values represent the percentage out of 21 participants for whom the prior, the outcome-bonus, or the prior+outcome-bonus model is better than the alternative models.
(DOC)

Table S3 Parameter values for the prior + outcome-bonus model.
(DOC)

Text S1 The text contains a detailed description of the computer simulation procedure and additional simulation results (see also Figures S2–S4). In addition, supporting materials and methods provide a detailed description of the experiment (see also Figure S4), behavioural results (see also Figure S5), all social learning models and modelling results (see also Tables S1 and S2), the model fitting procedure, and the fMRI analysis.
(DOC)

## Abbreviations

AIC: Aikake information criterion
BIC: Bayesian information criterion
BOLD: blood-oxygen level-dependent
fMRI: functional magnetic resonance imaging
MPFC: medial prefrontal cortex
OFC: orbitofrontal cortex
VMPFC: ventromedial prefrontal cortex
VST: ventral striatum

## References

[1] Bonaccio S, Dalal R (2006). Advice taking and decision-making: an integrative literature review, and implications for the organizational sciences.. Organizational Behavior and Human Performance.

[2] Boyd R, Richerson P (1985). Culture and the evolutionary process..

[3] Tomasello M, Carpenter M, Call J, Behne T, Moll H (2005). Understanding and sharing intentions: The origins of cultural cognition.. Behavioral and Brain Sciences.

[4] Henrich J, McElreath R (2003). The evolution of cultural evolution.. Evol Anthropol.

[5] Richerson P. J, Boyd R, Henrich J (2010). Colloquium paper: gene-culture coevolution in the age of genomics.. PNAS.

[6] Simon H (1990). A mechanism for social selection and successful altruism.. Science.

[7] Schotter A (2003). Decision making with naive advice.. American Economic Review.

[8] Capon N, Fitzsimons G, Prince R (1996). An individual level analysis of the mutual fund investment decision.. J Financ Serv Res.

[9] Duhan D, Johnson S, Wilcox J, Harrell G (1997). Influences on consumer use of word-of-mouth recommendation sources.. J Acad Market Sci.

[10] McElreath R, Bell A. V, Efferson C, Lubell M, Richerson P. J (2008). Beyond existence and aiming outside the laboratory: estimating frequency-dependent and pay-off-biased social learning strategies.. Philos Trans R Soc Lond, B, Biol Sci.

[11] Bikhchandani S, Hirshleifer D, Welch I (1992). A theory of fads, fashion, custom, and cultural change as informational cascades.. The Journal of Political Economy.

[12] Biele G, Rieskamp J, Gonzalez R (2009). Computational models for the combination of advice and individual learning.. Cognitive Science.

[13] Behrens T. E. J, Hunt L. T, Woolrich M. W, Rushworth M. F. S (2008). Associative learning of social value.. Nature.

[14] Klucharev V, Hytönen K, Rijpkema M, Smidts A, Fernández G (2009). Reinforcement learning signal predicts social conformity.. Neuron.

[15] Montague P. R, King-Casas B, Cohen J. D (2006). Imaging valuation models of human choice.. Annual Review of Neuroscience.

[16] Yechiam E, Busemeyer J. R (2005). Comparison of basic assumptions embedded in learning models for experience-based decision making.. Psychonomic Bulletin & Review.

[17] Montague P. R, Hyman S. E, Cohen J. D (2004). Computational roles for dopamine in behavioural control.. Nature.

[18] Doll B, Jacobs W, Sanfey A, Frank M. J (2009). Instructional control of reinforcement learning: a behavioral and neurocomputational investigation.. Brain Research.

[19] Schultz W (2006). Behavioral theories and the neurophysiology of reward.. Annu Rev Physiol.

[20] Kringelbach M. L (2005). The human orbitofrontal cortex: linking reward to hedonic experience.. Nat Rev Neurosci.

[21] O'Doherty J, Kringelbach M, Rolls E. T, Hornak J, Andrews C (2001). Abstract reward and punishment representations in the human orbitofrontal cortex.. Nat Neurosci.

[22] Knutson B, Cooper J (2005). Functional magnetic resonance imaging of reward prediction.. Current Opinion in Neurology.

[23] Tobler P. N, O'Doherty J. P, Dolan R. J, Schultz W (2007). Reward value coding distinct from risk attitude-related uncertainty coding in human reward systems.. Journal of Neurophysiology.

[24] Gottfried J. A, O'Doherty J, Dolan R. J (2003). Encoding predictive reward value in human amygdala and orbitofrontal cortex.. Science.

[25] Knutson B, Fong G, Adams C. M, Varner J, Hommer D (2001). Dissociation of reward anticipation and outcome with event-related fMRI.. Neuroreport.

[26] Krugel L. K, Biele G, Mohr P. N. C, Li S-C, Heekeren H. R (2009). Genetic variation in dopaminergic neuromodulation influences the ability to rapidly and flexibly adapt decisions.. PNAS.

[27] O'Doherty J, Dayan P, Schultz J, Deichmann R, Friston K. J (2004). Dissociable roles of ventral and dorsal striatum in instrumental conditioning.. Science.

[28] Olds J, Milner P (1954). Positive reinforcement produced by electrical stimulation of septal area and other regions of rat brain.. Journal of Comparative and Physiological Psychology.

[29] Powell E. W, Rorie D. K (1967). Septal projections to nuclei functioning in oxytocin release.. American Journal of Anatomy.

[30] Kosfeld M, Heinrichs M, Zak P. J, Fischbacher U, Fehr E (2005). Oxytocin increases trust in humans.. Nature.

[31] Wasserman L (2000). Bayesian model selection and model averaging.. Journal of Mathematical Psychology.

[32] Pessiglione M, Seymour B, Flandin G, Dolan R. J, Frith C. D (2006). Dopamine-dependent prediction errors underpin reward-seeking behaviour in humans.. Nature.

[33] Van Overwalle F (2009). Social cognition and the brain: a meta-analysis.. Human Brain Mapping.

[34] Krueger F, Mccabe K, Moll J, Kriegeskorte N, Zahn R (2007). Neural correlates of trust.. PNAS.

[35] Engelmann J. B, Capra C. M, Noussair C, Berns G. S (2009). Expert financial advice neurobiologically "Offloads" financial decision-making under risk.. PLoS ONE.

[36] Coricelli G, Dolan R. J, Sirigu A (2007). Brain, emotion and decision making: the paradigmatic example of regret.. Trends in Cognitive Sciences.

[37] Coricelli G, Critchley H. D, Joffily M, O'Doherty J. P, Sirigu A (2005). Regret and its avoidance: a neuroimaging study of choice behavior.. Nature Neuroscience.

[38] Adolphs R (2010). Conceptual challenges and directions for social neuroscience.. Neuron.

[39] Behrens T. E. J, Hunt L. T, Rushworth M. F. S (2009). The computation of social behavior.. Science.

[40] Winston J. S, Strange B. A, O'Doherty J, Dolan R. J (2002). Automatic and intentional brain responses during evaluation of trustworthiness of faces.. Nature Neuroscience.

[41] Koscik T. R, Tranel D (2010). The human amygdala is necessary for developing and expressing normal interpersonal trust.. Neuropsychologia.

[42] Niv Y, Schoenbaum G (2008). Dialogues on prediction errors.. Trends in Cognitive Sciences.

[43] Miller N, Dollard J (1941). Social learning and imitation..

[44] Sigmund K, De Silva H, Traulsen A, Hauert C (2010). Social learning promotes institutions for governing the commons.. Nature.

[45] Laland K. N, Odling-Smee J, Myles S (2010). How culture shaped the human genome: bringing genetics and the human sciences together.. Nature Reviews Genetic.

[46] Salganik M. J, Dodds P. S, Watts D. J (2006). Experimental study of inequality and unpredictability in an artificial cultural market.. Science.

[47] Luce R. D (1959). Individual choice behavior..

[48] Van Overwalle F (2009). Social cognition and the brain: a meta-analysis.. Human Brain Mapping.

[49] Krueger F, Mccabe K, Moll J, Kriegeskorte N, Zahn R (2007). Neural correlates of trust.. PNAS.

[50] Ridderinkhof K. R, Ullsperger M, Crone E. A, Nieuwenhuiss S (2004). The role of the medial frontal cortex in cognitive control.. Science.

