# Supplementary figures and images for: The Neural Basis of Following Advice
Source: PLoS Biol. 2011 Jun 21;9(6):e1001089. doi: 10.1371/journal.pbio.1001089 (PMC3119653; doi:10.1371/journal.pbio.1001089)

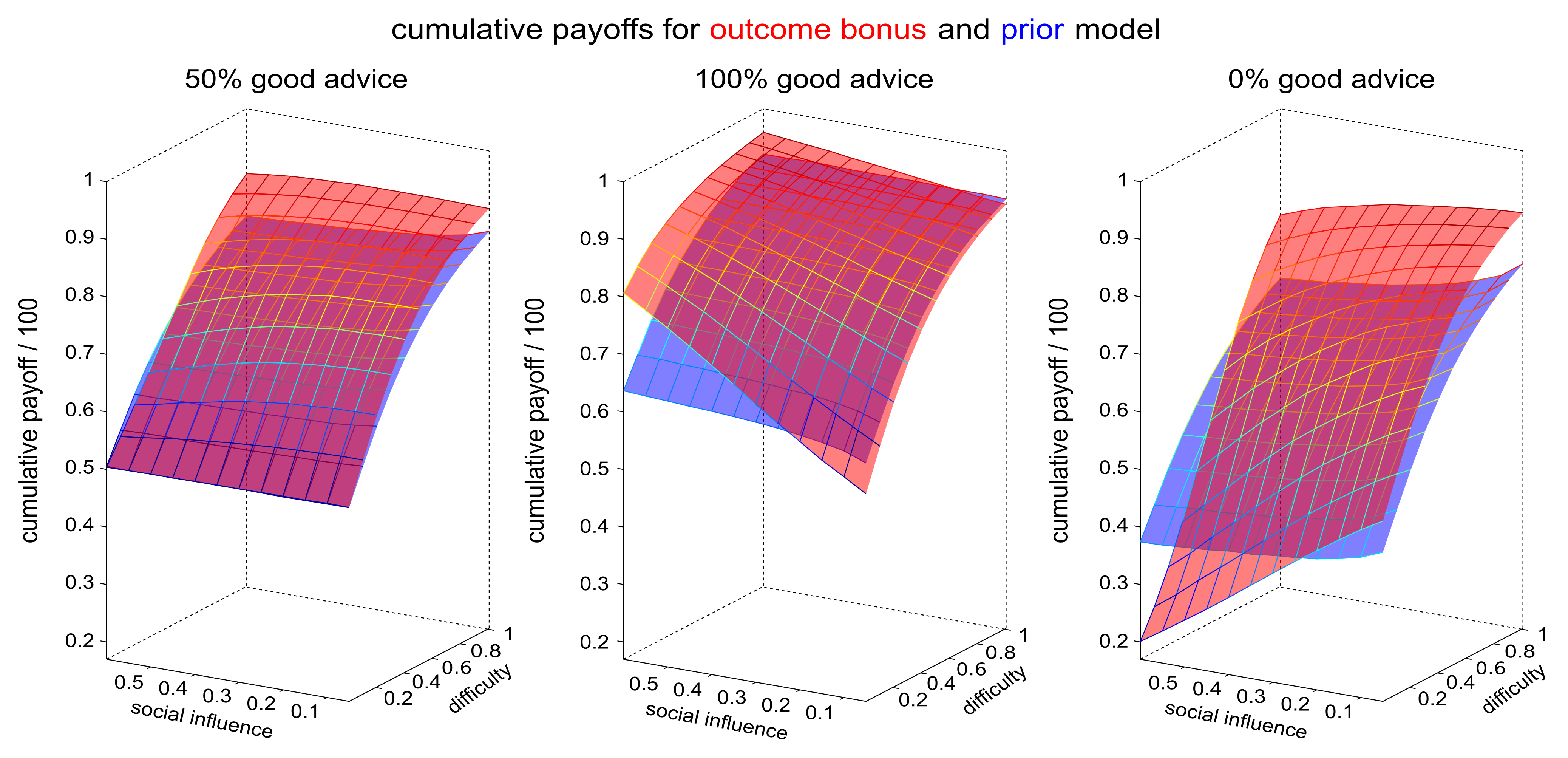

Supplement: Figure S1 — The payoff distribution for the four decks in the task. Good decks with higher average rewards were associated with relatively low gains and with moderate losses. Bad options with lower average rewards were associated with higher gains than the good decks, but also with much larger losses. (TIF) [file pbio.1001089.s001.tif]

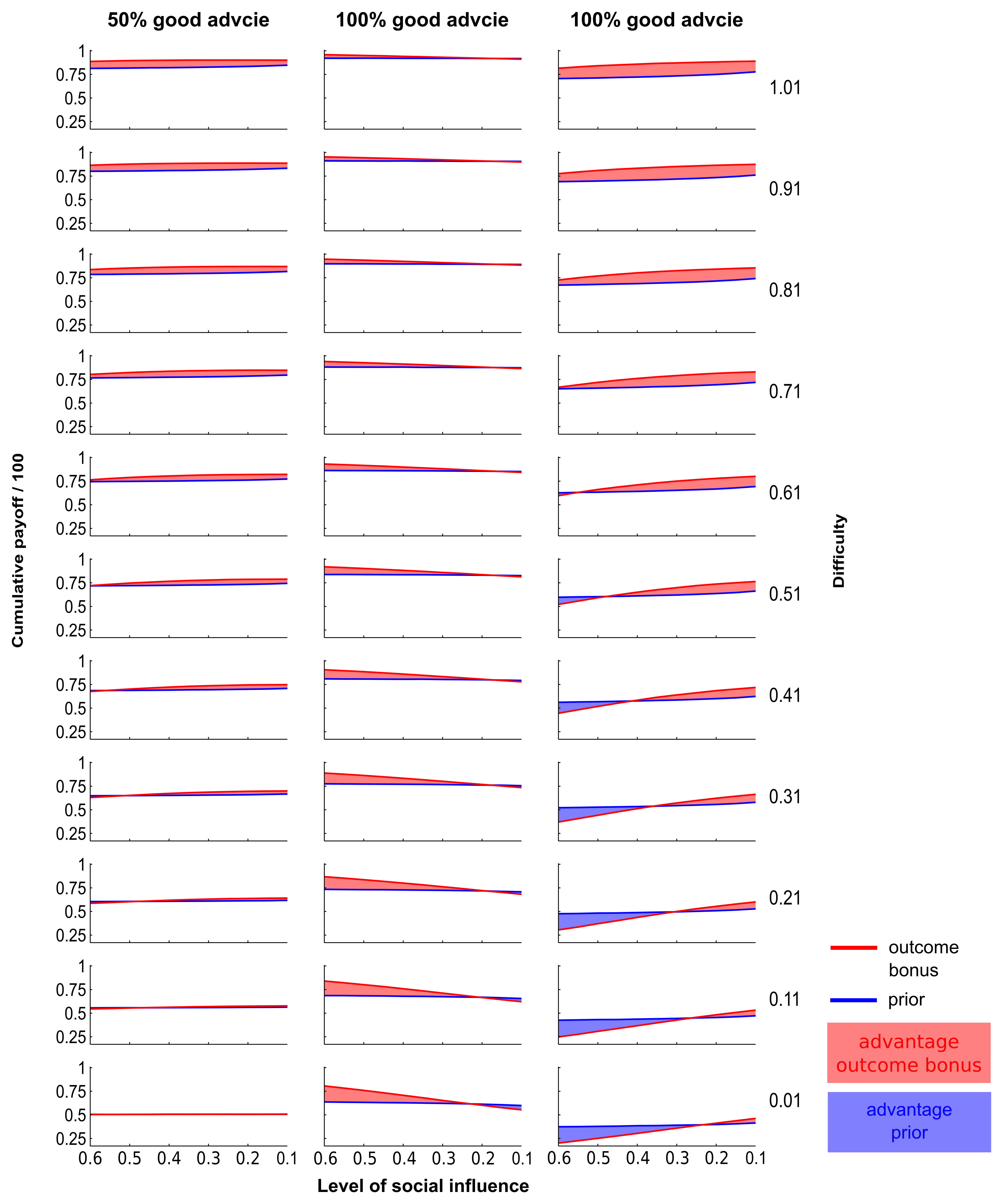

Supplement: Figure S2 — The probability of choosing one of the two good decks, separately for the first and second half of the experiment and for participants who received good and bad advice. Black lines indicate two standard errors of the mean. Participants who received bad advice (n = 5) generally chose the good decks less frequently than participants who received good advice (n = 16) but also improved performance from the first to the second half. (TIF) [file pbio.1001089.s002.tif]

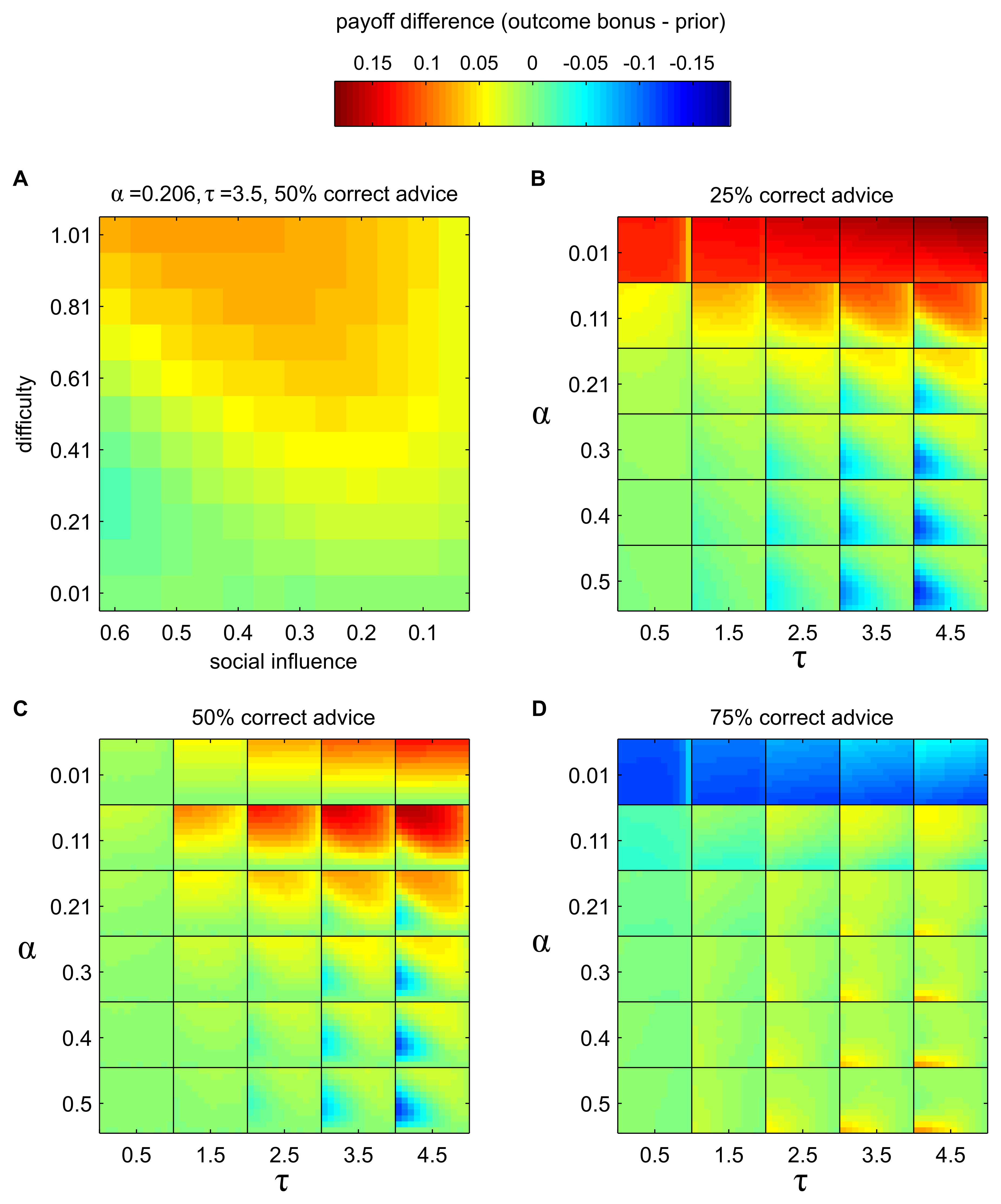

Supplement: Figure S3 — Performance of social learning models for typical learning parameters. Each panel shows one model's mean cumulative payoff on the z-axis. The levels of social influence and task difficulty are varied across the x- and y-axis, respectively. Difficulty is calculated as the difference in the mean payoff of the good and bad options, so that higher values indicate easier tasks. The performance of the outcome-bonus model is depicted by the blue surface, and the performance of the prior model is depicted by the red surface. (TIF) [file pbio.1001089.s003.tif]

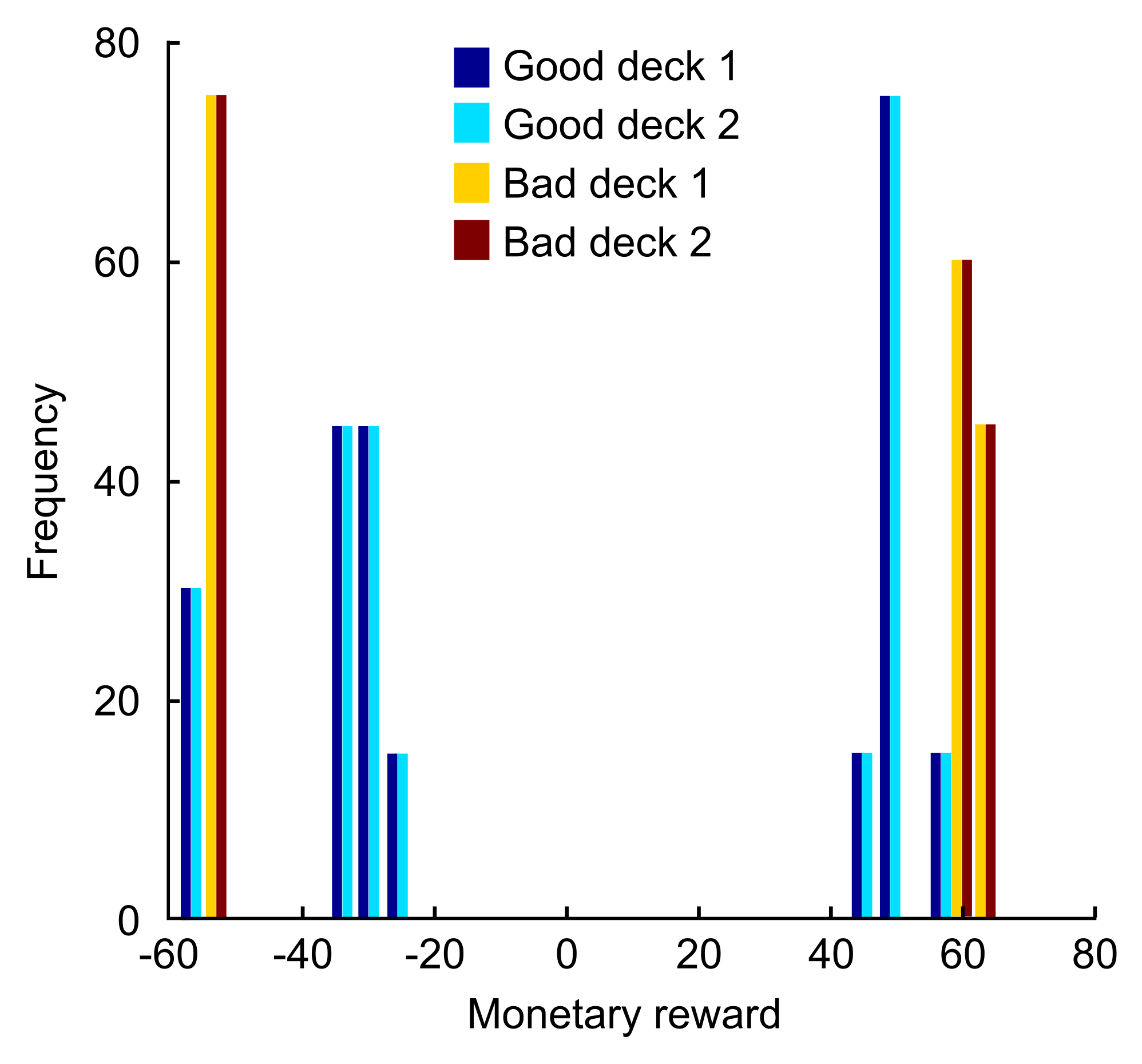

Supplement: Figure S4 — Performance of social learning models for typical learning parameters. (This figure presents the same data as Figure S3 in a different format.) Each subplot shows average payoffs of the two social learning models for different levels of social influence. The difficulty of the basic learning task (higher values indicate easier tasks) is varied along the global y-axis with easy tasks at the top and hard tasks at the bottom. The three columns show results for different qualities of advice. In sum, the figure indicates that the outcome-bonus model generally performs better. The prior model only performs better when, at the same time, (a) the task is hard, (b) bad advice is more likely, and (c) the social influence is relatively strong. The reason is that, in this case, the outcome-bonus can be larger than the payoff difference between good and bad options, so that the outcome-bonus model consistently prefers the recommended but bad option. (TIF) [file pbio.1001089.s004.tif]

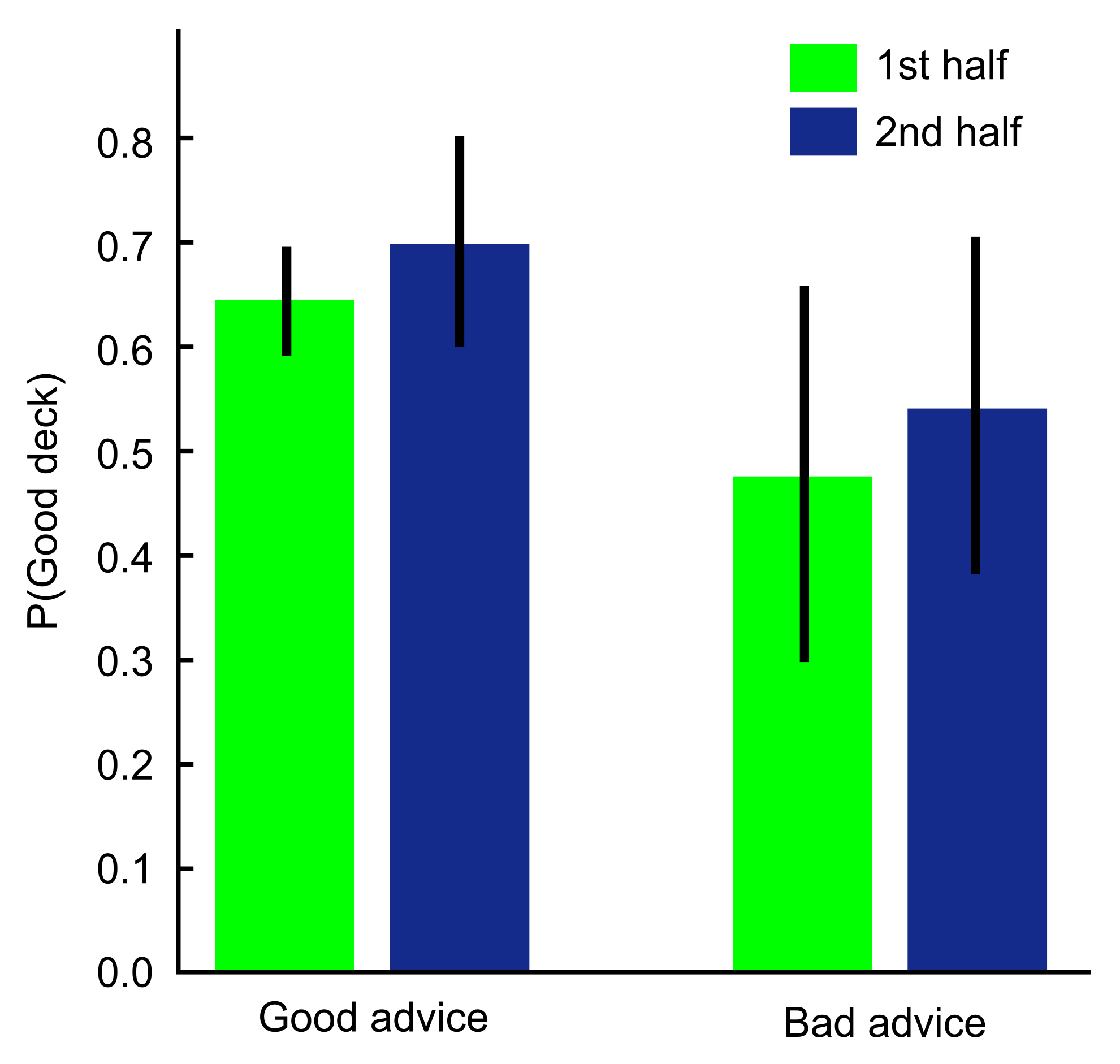

Supplement: Figure S5 — Performance differences in the social learning models across different learning parameters. Panel A shows the performance differences of the models. That is, the mean payoff of the outcome-bonus model minus the mean payoff of the prior model for typical learning parameters and 50% correct advice, while varying the impact of social influence (x-axis) and difficulty (y-axis). Panels C shows replications of the different difficulty and social learning parameters examined in Panel A for different learning rates (on the x-axis) and choice sensitivities (on the y-axis). Panels B and D show results of the same analysis for 25% and 75% good advice. Note that hotter colors (red and yellow) indicate an advantage of the outcome-bonus model, cooler colors (blue and cyan) indicate an advantage of the prior model, and neutral color (green) indicates similar performance of the two models. (TIF) [file pbio.1001089.s005.tif]

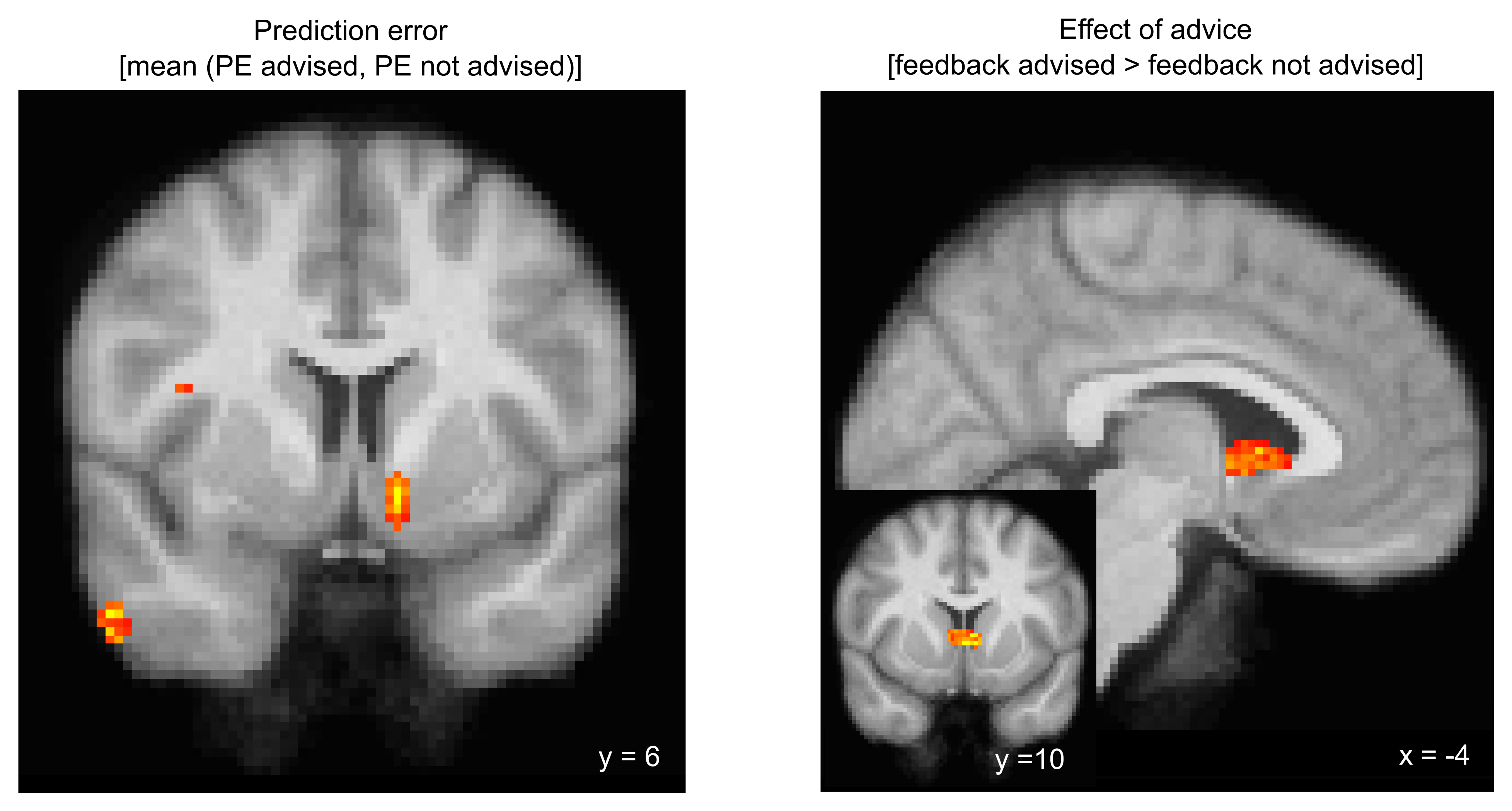

Supplement: Figure S6 — The results of an alternative fMRI analysis optimized to detect correlations of BOLD response and prediction errors. The left panel shows a region in the ventral striatum that correlates positively with prediction errors for recommended and non-recommended options (x = −10, y = 6, z = −8, max z-value = 3.1). The right panel shows the results of contrasting the intercept regressors for recommended minus non-recommended feedback (x = −4, y = 10, z = 4, max z-value = 3.2). The effect of advice on feedback signals identified in this analysis comprises the same region as identified in the original analysis and reported in the main text. (TIF) [file pbio.1001089.s006.tif]

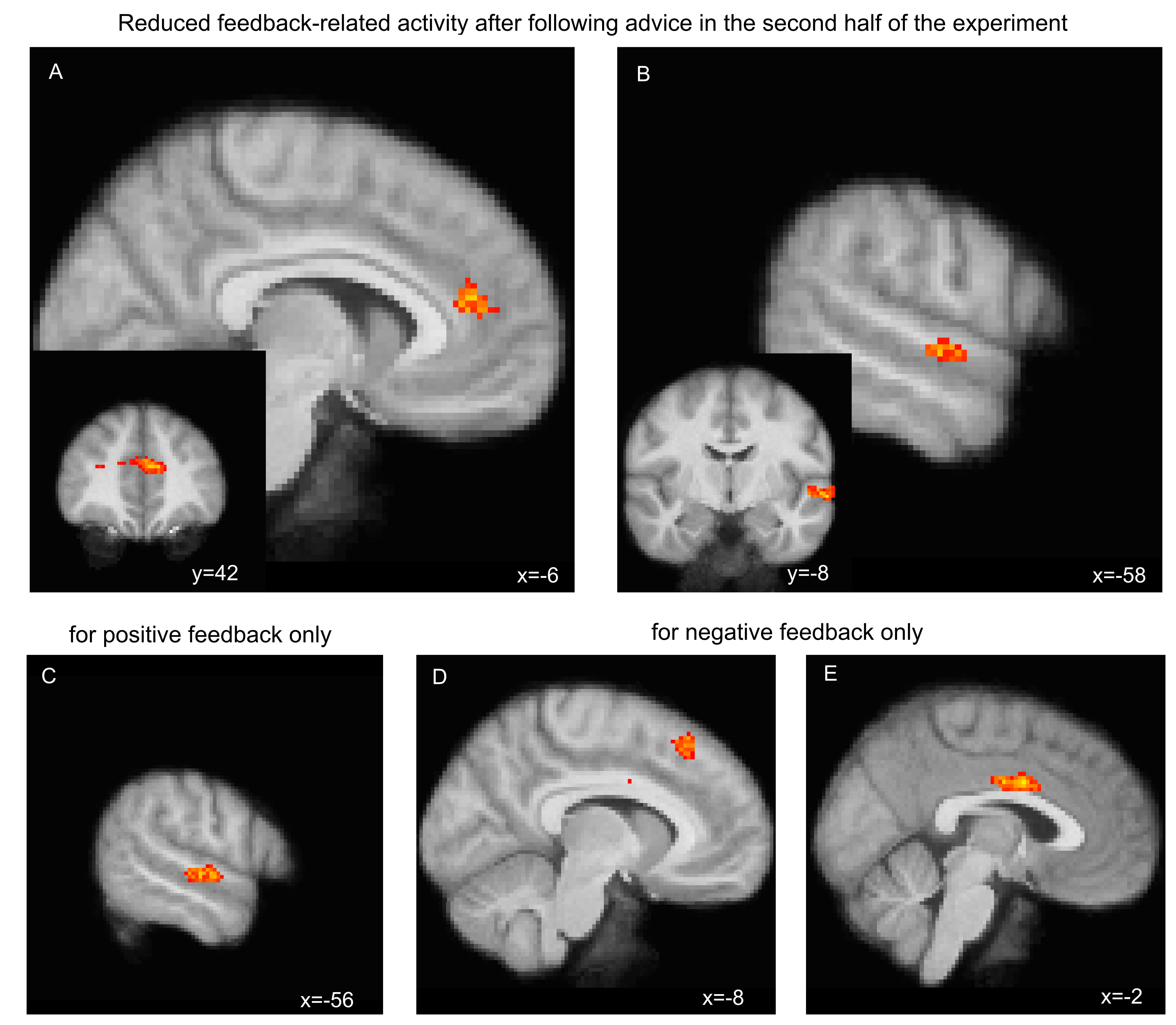

Supplement: Figure S7 — The results of a supplementary analysis, investigating potential changes in the outcome-bonus signal over time. This analysis was based on the original analysis, to which we added two more regressors capturing (f) gain feedback- and (g) loss feedback-related activity after following advice for the second half of trials, in which participants chose the recommended option. Importantly, in this analysis, we found the same outcome-bonus signal in the septal area as identified in the original analysis (shown in Figure S8). Parameter estimates for regressors (f) and (g) did not differ significantly from zero in regions implementing the outcome-bonus. Instead, we found a reduced BOLD signal for feedback in regions commonly associated with theory-of-mind (TOM) reasoning or negative feedback after following advice. These results are significant after whole brain correction, based on a z-threshold of 2.576 and a minimum cluster size of 152 voxels (cluster size criterion obtained with AFNI AlphaSim, see Text S1). (A) Reduced activation for feedback after following advice (i.e. the contrast vector had a −1 for regressors (f) and (g) and 0 for all other regressors) in the dorsomedial prefrontal cortex/paracingulate cortex (DMPFC/PCC, x = −6, y = 42, z = 16, max z-value = 3.13). (B) Reduced activation for feedback after following advice in the superior temporal sulcus (STS, x = −64, y = −8, z = −6, max z-value = 3.21). The PCC and STS are commonly associated with reasoning about the intentions and traits of other people and have been shown to be active when participants play economic games like the trust game [48],[49]. (C) Reduced activation for positive feedback after advice-following in the STS (x = −62, y = −6, z = −4, max z-value = 3.11). (D) Reduced activation for negative feedback after following advice in the rostral cingulate zone (RCZ, x = −20, y = 24, z = 48, max z-value = 3.21). The RCZ is associated with processes of conflict monitoring and learning from negative fee [file pbio.1001089.s007.tif]

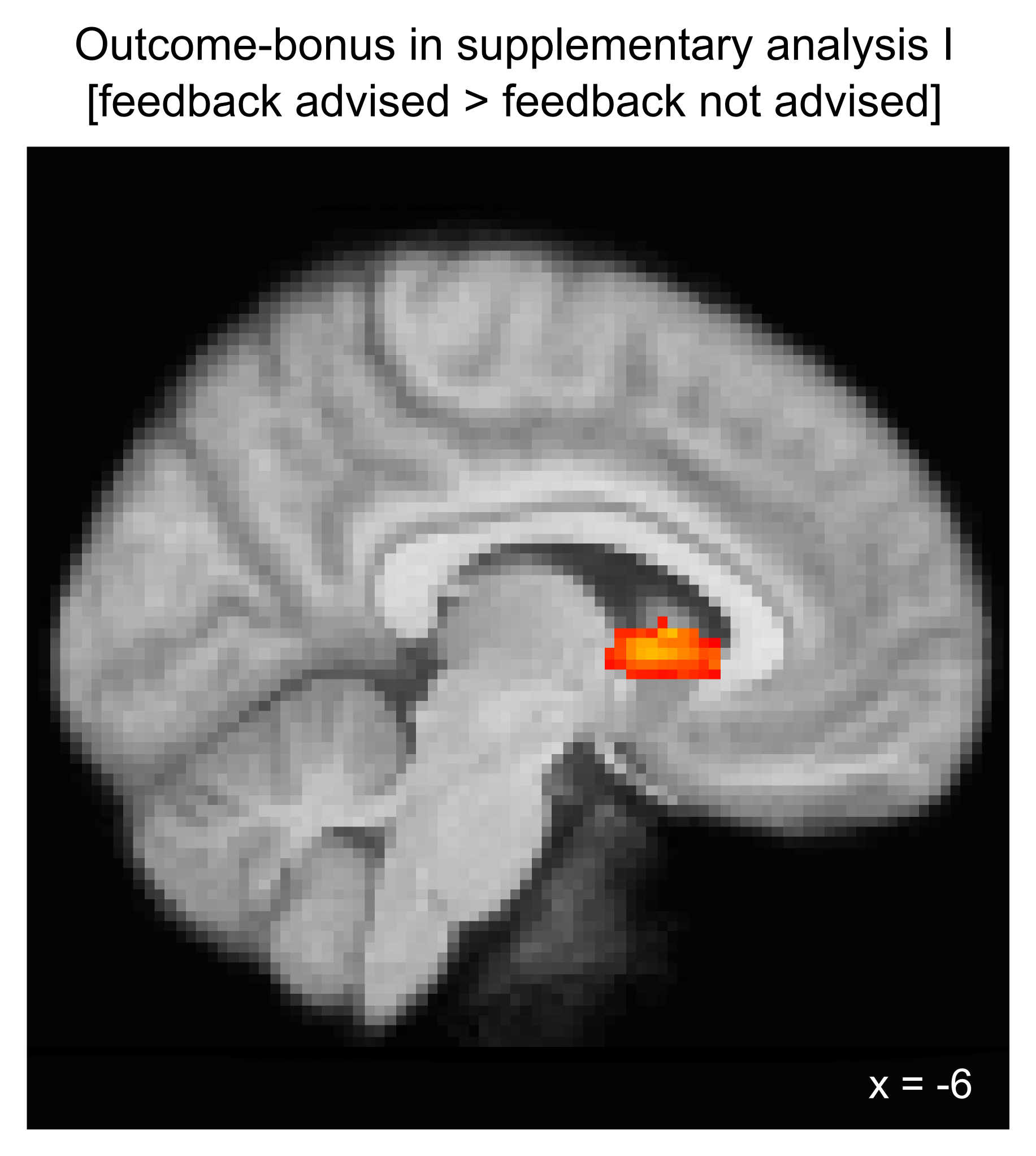

Supplement: Figure S8 — Result for the contrast of feedback advised > feedback not advised of the control analysis described in the legend to Figure S7. We still identified an outcome-bonus signal for feedback after following advice versus not following advice in the septal area/left caudate (x = −6, y = 12, z = 6, max z-value = 3.26) over the entire experiment, after controlling for the effects of time by introducing additional regressors modeling feedback effects for the second half of the experiment (details in the legend to Figure S7). (TIF) [file pbio.1001089.s008.tif]

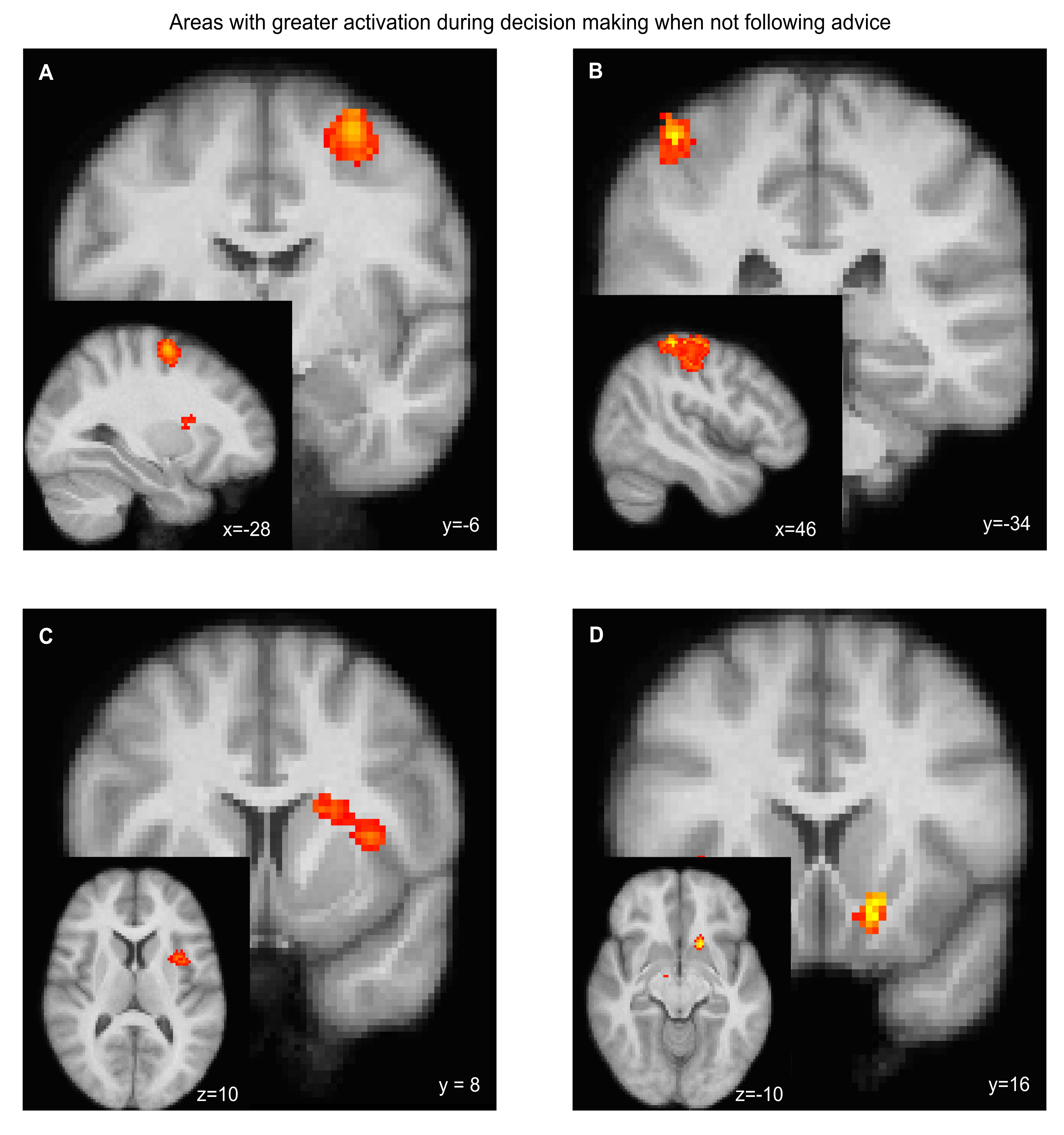

Supplement: Figure S9 — A number of regions showed greater activation when participants decided to not follow advice. (A) Left supplementary motor area (SMA, x = −28, y = −6, z = 60, max z-value = 3.33), (B) right post-central gyrus (x = 46, y = −34, z = 60, max z-value = 3.56), (C) left central operculum with extension into caudate and putamen (x = −34, y = 8, z = 10, max z-value = 3.05), (D) and left VST (x = −28, y = −6, z = 60, max z-value = 3.07). Results shown in (A, B, C) are significant after whole brain correction, based on a z-threshold of 2.576 and a minimum cluster size of 152 voxels (cluster size criterion obtained with AFNI AlphaSim). With a cluster size of 81 voxels, the VST result approaches significance when correcting for multiple comparison in the reward ROI (minimum cluster size would be 92 voxel). In particular, the reduced activation in the SMA and the VST suggests that following advice is accompanied by a reduction in decision-related brain activity. A similar result has been reported for following advice in the context of financial decisions [35]. (TIF) [file pbio.1001089.s009.tif]
