# Supplementary material for: The Neural Basis of Following Advice
Source: PLoS Biol. 2011 Jun 21;9(6):e1001089. doi: 10.1371/journal.pbio.1001089 (PMC3119653; doi:10.1371/journal.pbio.1001089)
Supplement: Table S1 — The table provides basic information about the compared models. Columns under the header “Implemented social learning mechanisms” contain a “+” if a particular model implemented the respective social learning mechanism. LL is the log likelihood, AIC is the Aikake information criterion, BIC is the Bayesian information criterion (see supplementary methods for details). (DOC) [file pbio.1001089.s010.doc]

Table S1. Overview of tested models

| Model name | Impleme | mented | social | learning | mechani | sm | # of free parameters | Model fit | and select | ion criteria |
| --- | --- | --- | --- | --- | --- | --- | --- | --- | --- | --- |
|  | prior | outcome-bonus | dynamic bonus | gain bonus | loss bonus | zero loss |  | LL | AIC | BIC |
| Individual learning |  |  |  |  |  |  | 2 | -4238 | 8476 | 8476 |
| Outcome-bonus |  | + |  |  |  |  | 3 | -4105 | 8253 | 8318 |
| Prior | + |  |  |  |  |  | 3 | -4137 | 8315 | 8381 |
| Prior+outcome-bonus | + | + |  |  |  |  | 4 | -4059 | 8201 | 8332 |
| Dynamic bonus |  |  | + |  |  |  | 4 | -4104 | 8291 | 8422 |
| Dynamic bonus+prior | + |  | + |  |  |  | 5 | -4044 | 8215 | 8411 |
| Gain bonus |  |  |  | + |  |  | 3 | -4125 | 8293 | 8358 |
| Loss bonus |  |  |  |  | + |  | 3 | -4134 | 8309 | 8375 |
| Zero Loss |  |  |  |  |  | + | 2 | -4207 | 8414 | 8414 |
