# Supplementary material for: The Neural Basis of Following Advice
Source: PLoS Biol. 2011 Jun 21;9(6):e1001089. doi: 10.1371/journal.pbio.1001089 (PMC3119653; doi:10.1371/journal.pbio.1001089)
Supplement: Table S2 — Values represent the percentage out of 21 participants for whom the prior, the outcome-bonus, or the prior+outcome-bonus model is better than the alternative models. (DOC) [file pbio.1001089.s011.doc]

Table S2. Model comparison on a participant-by-participant basis Comparison based on AIC Comparison based on BIC

|  | Comparison | based on AIC |  | Comparison | based on BIC |  |
| --- | --- | --- | --- | --- | --- | --- |
|  | Prior | Outcome-bonus | Prior+outcome-bonus | Prior | Outcome-bonus | Prior+outcome-bonus |
| Individual learning | 81 | 81 | 85.7 | 42.9 | 57.1 | 47.6 |
| Outcome-bonus | 54.5 | - | 47.6 | 54.5 | - | 19 |
| Prior | - | 45.5 | 47.6 | - | 45.5 | 28.6 |
| Prior+outcome-bonus | 52.4 | 52.4 | - | 71.4 | 81 | - |
| Dynamic bonus | 66.7 | 81 | 72.7 | 66.7 | 90.5 | 72.7 |
| Dynamic bonus+prior | 61.9 | 57.1 | 71.4 | 71.4 | 81 | 90.5 |
| Gain bonus | 59.1 | 68 | 71.4 | 59.1 | 68 | 33.3 |
| Loss bonus | 59.1 | 64 | 76.2 | 59.1 | 64 | 42.9 |
| Zero Loss | 52.4 | 61.9 | 81 | 42.9 | 42.9 | 42.9 |
| *Average* | 59.7 | 62.3 | 67.1 | 57.6 | 64.4 | 47.5 |
