# Supplementary material for: The Neural Basis of Following Advice
Source: PLoS Biol. 2011 Jun 21;9(6):e1001089. doi: 10.1371/journal.pbio.1001089 (PMC3119653; doi:10.1371/journal.pbio.1001089)
Supplement: Table S3 — Parameter values for the prior + outcome-bonus model. (DOC) [file pbio.1001089.s012.doc]

Table S3. Parameter values for the prior + outcome-bonus model.

| Parameter | Median | Inter-quartile range |
| --- | --- | --- |
| α | 0.12 | 0.73 |
| τ | 1.51 | 9.12 |
| β_p_ | 0.02 | 3.99 |
| β_b_ | 2.93 | 7.25 |
