# Supplementary material for: The Neural Basis of Following Advice
Source: PLoS Biol. 2011 Jun 21;9(6):e1001089. doi: 10.1371/journal.pbio.1001089 (PMC3119653; doi:10.1371/journal.pbio.1001089)
Supplement: Text S1 — The text contains a detailed description of the computer simulation procedure and additional simulation results (see also Figures S2–S4). In addition, supporting materials and methods provide a detailed description of the experiment (see also Figure S4), behavioural results (see also Figure S5), all social learning models and modelling results (see also Tables S1 and S2), the model fitting procedure, and the fMRI analysis. (DOC) [file pbio.1001089.s013.doc]

Supporting Information for

**The neural basis of following advice**

Please send correspondence to Guido Biele at g.p.biele@psykologi.uio.no

**Simulations**

From an adaptive perspective, the rewards accumulated during learning determine the success of a learning algorithm. For instance, imagine comparing the success of two foragers who can choose to feed from four food sources. Here, the relevant criterion is not which forager estimates the expected value of the four food sources more accurately but which forager collects more food from the sources. Interestingly, this optimality criterion is different from the one typically used in the assessment of learning algorithms, which is the deviation of the learned value (in our case, the expected rewards) from the criterion value (here, the true mean of the payoff distributions) when the learning algorithm has converged to a stable estimate [1].

Of course, estimation accuracy and the amount of obtained food will generally be correlated. However, dependent on how value estimations are translated into choice, this correlation does not need to be perfect. In particular, when probabilistic choice rules are at work (which is strongly suggested by the decision-making and learning literature, as in Herrnstein’s matching law or the softmax choice rule, for instance), so that the option with the higher expected value is not chosen with certainty, overestimating the expected reward for the best option can lead to the accumulation of more rewards than the accurate estimation of expected rewards. Overestimating the expected value of the best option enhances the contrast between decision options, and makes it easier to choose the best option. Hence, a learning mechanism that overestimates the expected reward from a recommended option, as the outcome-bonus model does after good advice, can lead to the best performance in terms of accumulated rewards.

This explanation implies that bad advice is harmful, because it reduces the separation of the best option from the falsely recommended option or even leads to an incorrect ranking of options. Crucially, however, the outcome-bonus model also copes better with bad advice than a strong but transient influence of advice on initial expectations, as in the prior model. The reason is that the strong transient influence of bad advice reduces to zero the probability for choosing the good options in the early learning period, thus erasing any positive effect of individual learning. Even though this negative effect is limited to the beginning of learning, it is so strong that it has a substantial influence on cumulative rewards.[[1]](#footnote-2)

The simulations used to compare the prior model and the outcome-bonus model are based on a reinforcement-learning model with a delta learning rule and a softmax choice rule (for detailed model descriptions, see the sub-section Behavioral Analysis in the section Materials and Methods in the main article). The learning task was a four-armed bandit task with two identical “good” and two identical “bad” options, where the payoffs for each option were drawn from a uniform distribution with a range of 1. In the simulations, three groups of parameters were varied: those relating to (a) the learning environment, (b) the basic learning algorithm, and (c) the extent of social learning. The learning environment was manipulated by changing the following:

- The task difficulty, i.e., the difference of mean payoffs of good and bad options (.01 to 1.01; easier tasks have higher values)
- Percent correct advice (0, 25, 50, 75, 100%)

The payoff distributions were generated in two steps. First, for each of the four options, 100 payoffs were drawn from a uniform distribution with a minimum value of 0.5 and a maximum value of 1.5. Second, for the two bad options, we subtracted half of the difficulty parameter value from each payoff, and for the two good options we added half of the difficulty parameter value to each payoff. The basic learning algorithm was manipulated by varying the following:

- The learning rate *α* for individual learning (.1 to .5) and
- The choice sensitivity (or 1/temperature) *τ* for the choice function (.5 to 4.5).

Finally, the extent of social learning was manipulated by varying the social influence parameter β (.05 to 1.2). The social influence parameter determines how strong the influence of advice is compared to the average task difficulty.

For the outcome-bonus model, social influence was determined to be the social influence parameter *β* times the mean value of the difficulty parameter across all environments (.51). To allow for the same maximum weight of social influence in both models, the social influence parameter for the prior model was determined to be *β* times the mean difficulty parameter value times the number of learning trials (*N* = 100). More specifically, when the prior value is equal to the outcome-bonus times the number of trials, and the learning rate is 0, then the sum of the social influence is the same for the outcome-bonus and prior models. In addition, the two constants (mean difficulty and number of trials) allow us to set plausible bounds on the level of social influence. An alternative approach would be to choose each model’s best social learning parameter. However, this approach would be questionable, as different social learning parameters are optimal for different probabilities of correct advice and different task difficulties, and it seems highly implausible that people would generally have this knowledge. By comparison, the approach we chose does not assume that people have precise knowledge about advice quality or the difficulty of specific tasks. Importantly, the key intuition that explains why the two models perform differently is independent of details like the precise extent of social influence.

Reflecting the design of our fMRI experiment, we evaluated the models by their performance in a situation in which the quality of advice is uncertain. We chose a probability of 50% good advice to represent this uncertainty, because this allows us to identify a model that is robust towards bad advice (in the sense that it does not suffer too much from it). To better understand the performance for 50% good advice, we also examine and discuss the results for only bad advice or only good advice. Figures S3 and S4 show the performance of the outcome-bonus and the prior models in case of 50%, 0%, and 100% good advice and for typical learning parameters (*α* = .2; τ = 3.5). The case of 50% good advice simulates the situation of a person that cannot be sure whether advice will be good.

Figures S3 and S4 indicate that the outcome-bonus model is superior for most difficulty levels and for most levels of social influence. In particular, when the chance of receiving good advice is 50%, there are only some cases (i.e., when the task is difficult, and social influence is large) for which the prior model performs better. The right panel in Figure S3 shows that this applies because the outcome-bonus model performs particularly poorly when bad advice is combined with strong social influence. In this case, it is impossible for the outcome-bonus model to learn the correct choice, because the outcome-bonus permanently erases the difference between the recommended bad option and the good options (and, in the extreme, makes the recommended bad option look better than the good options). In contrast, even strong initial social influence eventually decays for the prior model, so that the learner eventually chooses the better option.

The middle panel in Figure S3 and the second column in Figure S4 show that, when advice is always good and the influence of social learning is weak, the prior model outperforms the bonus model. This relates to the softmax choice rule, under which the small outcome-bonus does not change choice probabilities substantially. By comparison, the substantial initial advantage of the recommended option in the prior model assures that the better options are chosen with a very high probability in the first trials.

One particularly interesting result is that the outcome-bonus model is better than the prior model even after bad advice for small to moderate levels of social influence and tasks that are not very difficult (c.f., Figure S3, right panel and the right column in Figure S4). The explanation is that, while the outcome-bonus leads to a sustained influence on choice probabilities, this influence is relatively weak as long as the outcome-bonus is small compared to the difference between expected payoffs for good and bad options. By comparison, initial choices will be severely influenced in the prior model, which implements a strong impact of social information at the beginning of learning, even for smaller social influence parameters. As Figure 3B of the main article shows, it takes a substantial number of trials until social influence is unlearned, so that bad advice severely reduces accumulated rewards. One way to help the prior model achieve a better performance would be to have much weaker social influence, but then the prior model would also profit much less from good advice.

Figure S5 displays the performance difference between the two models for a variety of learning parameters and for different percentages of correct advice. Panel A corresponds to Figure 3A of the main article and shows the performance under typical learning parameters with 50% correct recommendations for different task difficulties and levels of social influence. Each small rectangle in Panels B, C, and D displays the payoff difference between the outcome-bonus and the prior model for a different combination of learning parameters. Panels B, C, and D assume that 25%, 50%, or 75% of the recommendations are correct, respectively. First, note that, for the majority of environments, the outcome-bonus model is either better than or equally successful as the prior model. Panels B and C show that the outcome-bonus model has a particular advantage when bad advice is relatively frequent and the learning rate is low. This is due to the strong impact of the (mostly wrong) high initial reward expectation for the recommended option, which decays only slowly in the prior model when the learning rate is small. For the same reason, the prior model performs well in combination with a small learning rate when advice is predominantly good. Further, Panels B and C show that the bad performance of the outcome-bonus model in difficult tasks with strong social influence is persistent as long as bad advice is relatively frequent. Finally, Panel D shows that when good advice is frequent (as it was in our fMRI experiment), the outcome-bonus model’s advantage is particularly high, if the learning rate is not very small and social influence is strong, because the effect of good advice will decay quickly in the prior model, whereas the effect of good advice will be sustained in the outcome-bonus model.

In sum, the following insights explain why the outcome-bonus model performs better overall than the prior model:

- When advice is good, the sustained effect of advice in the outcome-bonus model is superior to the transient effect implemented in the prior model. Overestimating the expected reward from the recommended option maximizes reward when probabilistic choice rules determine decisions.
- When advice is bad, the outcome-bonus suffers from it. However, this effect is only moderate, as long as the outcome-bonus is small compared to the payoff differences between good and bad options. Notably, the size of the outcome-bonus required to profit from good advice is still small enough to prevent bad advice from being harmful (c.f., Figure S5).
- In contrast, even relatively low levels of social influence can be harmful for the prior model in easy tasks, because it stops the learner from choosing the good option at the beginning of learning. Bad advice substantially harms the outcome-bonus model only in difficult tasks when the outcome-bonus is large compared to the difference in expected values between good and bad options.

**Materials and Methods**

*Participants*

For the fMRI experiment, 21 right-handed participants (ten women, eleven men; mean age 26 ± 2.6 years) were recruited from a participant database of the Max Planck Institute for Human Development. All participants were free of neurological and psychiatric history and gave informed consent to participate according to the protocol approved by the Charité University Medicine, Berlin. An additional ten participants were recruited to function as advisors for participants in the fMRI experiment.

*Experiment*

Participants performed a four-armed bandit task, in which they made 168 choices from four virtual card decks representing the four bandits. Participants were encouraged to perform as well as possible, which was emphasized by providing a bonus payment dependent on performance. To make the learning task difficult so that the advice of others would be useful, the two good decks led to small gains and comparatively smaller losses, whereas the bad decks led to large gains and comparatively larger losses. Specifically, each of the decks was associated with the following payoff distributions (see Figure S1): The two “good” options had a high expected payoff of 0.10 €. From each good deck, 50% of the payoffs were positive with a mean of 50 cents (SD = 2.8), and 50% were negative with a mean of -30 cents (SD = 2.8). The two “bad” decks had a low expected payoff of 0.025 €. From each bad deck, 50% of the payoffs were positive with a mean of 60 cents (SD = 2.8), and 50% were negative with a mean of -55 cents (SD = 2.8). Participants were informed that they would lose 25 cents for each trial in which they failed to choose any option.

Each trial began with the presentation of four card decks on the screen for 2.5 s, during which participants had to choose one deck. Participant indicated their choice by pressing one of four buttons on response box they hold with their right hand. After the presentation of the card decks, a fixation cross was presented for a variable time (M = 3.7 sec, Range: 1-16 sec) determined with *optseq2* (http://surfer.nmr.mgh.harvard.edu/optseq/) [2]. After the fixation cross, feedback was presented for 2.5 s, indicating how many cents the participant had won or lost in the current trial. No information about cumulative payoffs was provided. Each trial ended with the presentation of another fixation cross for a variable time period (M = 3.75 sec, Range: 2.5-7.5 sec). The experiment comprised a total of 168 trials (6 runs of 28 trials).

We used *Presentation* (Neurobehavioral Systems, San Pablo, USA) to present the stimuli and to record the responses on a notebook with Windows XP (Microsoft, Seattle, USA) as the operating system. Visual stimuli were presented using an LCD Projector and a mirror system.

Prior to the fMRI experiment, a different group of ten participants performed the task as advisors in a behavioral experiment. These participants were informed at the beginning of the session that they would be the advisor for another participant performing the same task later in an fMRI experiment and that they would receive a payment equal to 50% of the advice receiver’s earnings (in addition to their earnings from their own learning task). The advisor’s payment depended on the advice receiver’s performance and was paid after the advice receiver performed the task in the scanner. To give advice, advisors marked one of four recommendations on a form. Possible recommendations were “It is best to choose from deck A”, “… B”, “… C”, or “… D” (In German: “Wähle am besten von Deck A”, etc.).

For each advice receiver, one of the ten advisors was randomly selected. In the fMRI experiment each advice receiver was given one of the advice forms after reading the general instructions for the four-armed bandit task. Advice receivers were also informed that the advisor had performed the task prior to giving advice, and that the advisor would receive an additional payment equal to 50% of the advice receiver’s earnings. Thus, the advice receivers knew that the advisors had an incentive to provide good advice.

Participants in the fMRI experiment received a 20€ flat payment plus a bonus payment depending on their task performance (M = 11.7€, SD = 2.8).

*Behavioral analysis*

*Good vs. bad advice*: Participants were influenced by both advice and their individual learning experiences, regardless of whether advice was good or bad. Figure S2 shows that, throughout the experiment, participants who received good advice were more likely to choose one of the two good decks. The probability of choosing a good deck increased from the first to the second half of the experiment for both receivers of good advice (*p* < .0001) and receivers of bad advice (*p* = .0625). Due to the small sample size of participants with bad advice, we used non-parametric tests for the analysis of choice data.

*Models of social learning*: To investigate the influence of advice on learning, we compared a standard reinforcement learning model, an ‘outcome-bonus’ model, a ‘prior’ model, and a combined “prior +outcome-bonus ” model in predicting participants’ choices. In addition to the original outcome-bonus model, we also tested variations that assumed that the outcome-bonus is selective to either gains or losses, that losses are treated as zero outcomes, or that the outcome-bonus is dynamic over time (also in combination with the prior model).

The standard reinforcement learning model assigns each option *i* an expected reward *q_i_*(*t*). On the basis of the expected rewards, choices are made according to the softmax choice rule, which determines the probability p*_i_*(*t*) of choosing option *i* in round *t* as:

, (S1)

where *τ* is a sensitivity parameter determining how likely the deck with the largest expected reward will be chosen. Note that this choice function holds for all trials except the first, for which we assume that the decision maker chooses the recommended option. This assumption was implemented in all test models.

After a choice was made, and feedback was given, the expected rewards were updated on the basis of the prediction error, that is, the deviation between the expected and actually received reward as follows:

(S2)

where *r_i_*(*t*) is the reward obtained from choosing deck *i* in trial *t*, and α is the learning rate that determines the impact of the prediction error in the updating process.

The prior model assumes a higher initial reward expectation for the recommended choice option. Hence, the initial reward expectation in the prior model is defined as follows:

. (S3)

*N* is the number of trials in the learning experiment, which we chose as a simple scaling factor that allows us to compare the weight of the prior compared to the payoff that can be obtained in the experiment (we do not assume that participants precisely know *N*, although they probably have a good idea about the order of magnitude of the number of trials). μ is the expected payoff from choosing randomly and serves as a normalization constant to allow comparison across tasks with different payoffs.

The outcome-bonus model differs from the standard reinforcement-learning model by changing the reinforcement of outcomes from recommended options. Accordingly, the updating rule (Equation S2) was modified such that, when the recommended option was chosen, a constant bonus was added to the objective reward as follows:

(S4)

where *g*(*i*) is an indicator function that takes the value 1 if option *i* is recommended and the value 0 if option *i* is not recommended; *β_b_* is a free advice-bonus parameter. For the combined prior+outcome-bonus model, both the initial reward expectation and the outcome-bonus were used to modify the evaluation of the choice options.

We also tested whether the influence of advice on outcome evaluation depended on whether the outcome was positive (gain) or negative (loss) by implementing simple modifications of the outcome-bonus model: The gain-bonus model adds the outcome-bonus only if advice was followed and participants won money, the loss-bonus model adds the outcome-bonus only if advice was followed and participants lost money, and the zero-loss model treats all losses after following advice as zero rewards.

An alternative assumption is that the outcome-bonus is not stable but instead varies over time. We tested a dynamic outcome-bonus, which increases when advice turns out to be good and decreases when advice turns out to be bad. In this model, the outcome-bonus was calculated as follows:

(S5)

Here, *d* is a leaky accumulator that integrates the result of the comparison of payoffs received from the recommended option with payoffs received from the other options. *d* has the value 1 in the first trial and is updated according to the following equation:

(S6)

where *q_NR_* is the average payoff of not-recommended options, and *λ* is a leakage parameter that was estimated independently of the learning rate *α*. We deliberately chose a different learning rule rather than the delta learning rule, in which the learned quantities converge if *α* is small. By comparison, our model assumes that ‘trust’ or ‘distrust’ in advice continuously increases. That is, the longer a participant has experienced good advice, the greater the effective outcome bonus becomes. When the participant continuously experiences bad advice, the outcome-bonus will decrease and can eventually become negative (i.e., an advice malus or discount develops). We also estimated models that use the same learning rate for the dynamic outcome-bonus as for the general learning rule (i.e., *λ* = *α*). The theoretical motivation here was that participants who quickly (or slowly) adjust to expected rewards also quickly (or slowly) adjust the outcome-bonus. However, despite this additional parameter, the dynamic outcome-bonus models with separate learning rate and leakage parameter performed better than models that assume the same learning rate (i.e., where *λ* = *α*).

Finally, we also tested a model implementing the influence of advice on prior evaluation and the dynamic outcome-bonus. For this model, Equation S6 is replaced by the following equation, which removes the effect of advice-dependent priors while calculating the payoff difference between the recommended and the alternative options:

(S7)

Model parameters for all models were estimated for each participant’s learning data individually. As a goodness-of-fit measure, we used the log likelihood of the observed choices over all trials *N* given the model and its parameters: , where *f_t_*(*y*|θ) denotes the probability of choice *y* in trial *t*, given the model’s parameter set θ. The best model parameters were estimated on the basis of a “one step ahead” prediction; that is, the learning algorithm was applied to the observed choice history up to trial *t*-1 to generate the predicted choice of trial *t*. We determined the best-fitting parameters by first performing a grid search and using the best parameter combinations from the grid search as starting values for optimization with the Nelder-Mead Simplex algorithm [4]. The parameter values were constrained as follows: 0 < *α* < 1, 0.001 < *τ*, and 0 < *β* < 10. To compare the models, we used the Akaike information criterion (AIC) [5] and the Bayesian information criterion (BIC), which penalize models with more parameters than the baseline model: AIC = −2∙LL_m_+2∙d and BIC = −2∙LL_m_+log(*n*)∙d, where LL_m_ is the log likelihood of model *m*, *d* is the difference in the number of parameters for the tested model and the baseline model (which is the individual learning model), and *n* is the number of learning trials. We calculated the Bayes factor (BF) in favor of the first model_1 compared to the second model_2 using the BIC values with the formula BF = exp((BIC(model_1)-BIC(model_2))/-2 ) [6].

An overview of the models’ fits and the model selection criteria is given in Table S1. Considering the AIC and BIC/BF values together, the prior+outcome-bonus model is the best one, because only this and the outcome-bonus model are among the top three models when using AIC or BIC as selection criteria. These two models essentially perform equally well when comparing them based on BF, but the prior+outcome-bonus model is clearly better when compared to the outcome-bonus model with respect to AIC values. Table S2 shows how often the prior model, the outcome-bonus model, or the prior+outcome-bonus is the best model when compared on a participant-by-participant basis with the other eight models. In this analysis, the prior+outcome-bonus model performs best when the comparison is based on AICs but worse when the comparison is based on BICs, reflecting the greater imposed penalty for larger numbers of parameters in the BIC. Table S3 reports the best-fitting parameters for the prior+outcome-bonus model.

Note that removing the assumption of a different initial evaluation from the prior+outcome-bonus model increases the AIC value by only 52, whereas removing the assumption of an outcome-bonus increases the AIC value by 114, indicating that the outcome-bonus is more important to explaining the behavioral data than the higher initial expectation for the recommended option. Moreover, Table S2 shows that, in the prior+outcome-bonus model, the median parameter value for the outcome-bonus parameter is higher compared to the prior parameter for the initial evaluation of the recommended option (we set up the models so that these parameters are roughly on the same scale and are thus roughly comparable). These results strongly suggest that the outcome-bonus is the most important feature of a model explaining social learning.

*MRI data acquisition*

MRI data were acquired using a 1.5T Siemens Sonata (Erlangen, Germany) MRI scanner at Charité University Medicine, Berlin. We acquired six runs of 172 T2*-weighted echoplanar images (EPI) [TR, 25 ms; echo time (TE), 40 ms; flip angle, 90°; field of view, 256 mm; matrix, 64 x 64 mm; 26 axial slices approximately parallel to the bicommissural plane; slice thickness, 4 mm]. Three additional volumes were discarded at the beginning of each run to remove images with T1 equilibrium effects. In addition, for registration purposes, a T2-weighted matched-bandwidth high-resolution anatomical scan (same slice prescription as EPI) and a high-resolution T1-weighted structural image were acquired for each participant [TR, 20 ms; TE,5 ms; flip angle, 30°; 179 sagittal slices; voxel size, 1 x 1 x 1 mm].

*Image processing and registration:* Initial analysis was performed using the FSL toolbox from the Oxford Centre for fMRI of the Brain (FMRIB, www.fmrib.ox.ac.uk/fsl). The image time-course was first realigned to compensate for small head movements [7]. Data were spatially smoothed using an 8 mm full-width-half-maximum Gaussian kernel. Registration was conducted through a three-step procedure, whereby EPI images were first registered to the matched-bandwidth (T2) high-resolution structural image, then to the MPRAGE structural image (T1), and finally into standard Montreal Neurological Institute (MNI) space (MNI avg152 template), using 12-parameter affine transformations [8]. Statistical analyses were performed in native space, with the statistical maps normalized to standard space prior to higher-level analysis.

*Statistical analysis:* Statistical analysis of functional data was performed using a multi-level approach, implementing a mixed-effects model that treated participants as a random effect. Statistical modeling was first performed separately for each participant’s concatenated runs. Each run was denoised (including high-pass temporal filtering (Gaussian-weighted LSF straight line fitting, with sigma = 100s) and spatial smoothing with full width half maximum = 8mm) based on the results of FSL’s probabilistic independent component analysis MELODIC [9] with an automatic in-house procedure before concatenation.

Regressors of interest were created by convolving a rectangular function that represented stimulus duration times with a canonical (double-gamma) hemodynamic response function. Time-series statistical analysis was carried out using FILM (FMRIB's Improved Linear Model) with local autocorrelation correction [10].

The functional analysis was based on 12 regressors (plus six motion-parameter regressors): Two regressors modeled the choice of the recommended and the non-recommended option(s). The decision-related brain activity was assumed to start one second before the response and to continue until the response. Four partially overlapping binary regressors modeled positive and negative feedback after choosing the recommended and non-recommended options, respectively (duration: 2.5 sec). This property of the design matrix allowed us to independently investigate the influence of advice and of feedback valence on BOLD responses. An additional set of four corresponding parametric regressors controlled for feedback magnitude. One regressor modeled prediction errors estimated with the combined prior+outcome-bonus model. One error regressor modeled feedback after missed trials, in which participants made no decisions. Consistent with the concatenation procedure for the fMRI data, convolved regressors were high-pass filtered for each run separately before concatenation. For group-level results, individual level contrasts were averaged using the FMRIB Local Analysis of Mixed Effects (FLAME1+2, including automatic de-weighting of outliers) module in FSL [11,12], and one-sample t-tests were performed at each voxel for each contrast of interest.

Following our prediction that advice influences neural reward signals, we investigated representations of reward in a set of anatomically defined regions comprising the major reward-representing regions. We defined the reward regions based on the Harvard*-*Oxford subcortical structural atlas. The atlas’s probability maps were used to determine those voxels that had at least a 15% probability of belonging to one of the following anatomical regions: nucleus accumbens, caudate, putamen, thalamus, medial frontal cortex, and amygdala. Based on these probability maps, we created two binary maps, one for the amygdala and one for all other regions, which were also median-smoothed with a 15 mm sphere kernel. For the amygdala, *Z* (Gaussianized *T*) statistic images were thresholded with a small volume correction determined by *z* > 2.576 and a minimum cluster size of 36 voxels as determined with the AFNI AlphaSim tool [13]. For all other regions, *Z* statistic images were thresholded with a small volume correction determined by z > 2.576 and a minimum cluster size of 92 voxels, also determined with the AFNI AlphaSim tool.

*Analyses of Cluster Maxima*: Regions of interest (ROIs) were defined as spheres with a radius of 10 mm around the peak voxel of a significant cluster. For each ROI, parameter estimates and corresponding standard errors were calculated from the mean parameter estimates and mean variances of the ROI.

*Analysis of prediction error signals*: The design matrix for this analysis contained two regressors for choosing the recommended vs. non-recommended option(s). Feedback was modeled with four regressors altogether: one intercept regressor and one prediction error regressor for feedback after following and not following advice, respectively. The design matrix was completed with one error regressor capturing trials with no response and six motion parameter regressors.

**References for Supplementary Information**

1. Sutton RS, Barto AG (1998) Reinforcement learning: An introduction. Cambridge, Mass.: MIT Press.

2. Dale A (1999) Optimal experimental design for event-related fMRI. Hum Brain Mapp 8: 109-114.

3. Luce RD (1959) Individual choice behavior. Oxford, England: John Wiley.

4. Nelder JA, Mead R (1965) A Simplex-Method for Function Minimization. Computer Journal 7: 308-313.

5. Bozdogan H (2000) Akaike's information criterion and recent developments in information complexity. Journal of Mathematical Psychology 44: 62-91.

6. Wasserman L (2000) Bayesian model selection and model averaging. Journal of Mathematical Psychology 44: 92-107.

7. Jenkinson M, Bannister P, Brady M, Smith S (2002) Improved optimization for the robust and accurate linear registration and motion correction of brain images. NeuroImage 17: 825-841.

8. Jenkinson M, Smith S (2001) A global optimisation method for robust affine registration of brain images. Medical Image Analysis 5: 143-156.

9. Beckmann CF, Smith SM (2004) Probabilistic independent component analysis for functional magnetic resonance imaging. IEEE Transactions on Medical Imaging 23: 137-152.

10. Woolrich MW, Ripley BD, Brady M, Smith SM (2001) Temporal autocorrelation in univariate linear modeling of FMRI data. NeuroImage 14: 1370-1386.

11. Beckmann CF, Jenkinson M, Smith SM (2003) General multilevel linear modeling for group analysis in FMRI. NeuroImage 20: 1052-1063.

12. Woolrich MW, Behrens TEJ, Beckmann CF, Jenkinson M, Smith SM (2004) Multilevel linear modelling for FMRI group analysis using Bayesian inference. NeuroImage 21: 1732-1747.

13. Ward BD (2000) Simultaneous inference for fMRI data. Biophysics Reseach Institute, Medical College of Wisconsin.

1. As an example, consider a situation in which individual learning leads to choosing the good option in 70% of the first 20 trials. When advice is good, the prior model will choose the recommended-good option in 100% of the first 20 trials, an *improvement of 30* *percentage points*. However, when advice is bad, the prior model will choose the recommended bad option in 0% of the first 20 trials, a *decline of 70 percentage points*. Hence, for the prior model, the effect of bad advice is large compared to the effect good advice. [↑](#footnote-ref-2)
